# Increased BMD in SLD Patients Without Advanced Hepatic Fibrosis: Evidence From the NHANES 2017–2020 Database

**DOI:** 10.1155/cjgh/6969761

**Published:** 2025-08-11

**Authors:** Tianhao Wu, Lu Li, Yayuan Mei, Peizhen Lv, Jiawei Cui, Lin Liu, Yuemin Nan, Ang Li

**Affiliations:** ^1^Department of Orthopedic Surgery, Hebei Medical University Third Hospital, Shijiazhuang 050051, Hebei, China; ^2^Department of Gastroenterology, The Second Hospital of Tianjin Medical University, Tianjin 300211, China; ^3^Big Data Center, National Center for Children's Health, Beijing Children's Hospital, Capital Medical University, Beijing 100046, China; ^4^Department of Traditional and Western Medical Hepatology, Hebei Medical University Third Hospital, Shijiazhuang 050051, Hebei, China; ^5^Administrative Office, Hebei Orthopaedic Institute, Shijiazhuang 050051, Hebei, China; ^6^Department of Epidemiology and Biostatistics, Institute of Basic Medical Sciences Chinese Academy of Medical Sciences, School of Basic Medicine, Peking Union Medical College 100005, Beijing, China

**Keywords:** bone mineral density, cross-sectional study, hepatic fibrosis, NHANES, steatotic liver disease

## Abstract

**Background:** Recently, metabolic dysfunction-associated steatotic liver disease (MASLD) has been proposed to replace the liver condition previously known as nonalcoholic fatty liver disease (NAFLD), thereby redefining the subcategories of steatotic liver disease (SLD). However, the clinical relevance of SLD subcategories and their relationship with bone mass is lacking. In this study, we aimed to explore the potential association between the commonly proposed subclasses of fatty liver disease and bone mass.

**Methods:** A cross-sectional study using the data from the 2017–2020 cycle of the National Health and Nutrition Examination Survey (NHANES), involving 4237 participants aged 18 years and older who underwent vibration-controlled transient elastography (VCTE) and dual-energy X-ray absorptiometry (DXA), was conducted. A weighted generalized linear model was used to analyze the association of the SLD subcategories and bone mass changes including bone mineral content (BMC), bone area, and bone mineral density (BMD) in the femur and spine, with adjustments for potential covariates. Furthermore, a weighted generalized additive model was employed to assess the dose–response relationships between controlled attenuation parameter (CAP), liver stiffness measurement (LSM), and BMD.

**Results:** A total of 2635 and 1602 participants were included for analysis of the femur and lumbar spine, respectively. Compared to healthy individuals, positive correlations were observed between all three SLD subgroups (MASLD, MetALD, and ALD) and BMC, and BMD in the femur and spine, but no association with the bone area was identified. Moreover, CAP exhibited a strong positive correlation with BMD across all femoral and spinal scan sites. It was also positively correlated with BMC in some femoral scan sites and all spinal scan sites but was associated with the bone area only in certain femoral scan sites and not in spinal scan sites. In contrast, LSM showed clear positive correlations with BMD in some femoral and all spinal scan sites, as well as with BMC in certain femoral and spinal scan sites. However, LSM did not correlate with the bone area in any femoral or spinal scan sites. Besides, LSM showed a nonlinear association with these indicators. Subgroup analysis revealed a positive correlation between CAP and BMD only in individuals with CAP > 248 dB/m, BMI ≥ 25 kg/m^2^, and LSM < 11.7 kPa. Additionally, in females and individuals with LSM < 11.7 kPa, LSM was positively correlated with BMD, whereas in those with LSM ≥ 11.7 kPa, LSM showed a negative correlation with BMD.

**Conclusions:** Our findings highlighted a positive association between SLD and BMD; however, the association was likely influenced by liver fibrosis. Studies in large scale cohorts with a longer follow-up are warranted to elucidate the impacts of hepatic steatosis and associated pathologies on bone health.

## 1. Introduction

Nonalcoholic fatty liver disease (NAFLD) has rapidly emerged as the most common type of chronic liver disease (CLD) affecting approximately one-third of the world's population [1]. Although the clinical and histological features of NAFLD are liver-oriented [2], this condition is increasingly recognized as a multisystem disease with a close link to multiple metabolic disorders such as Type 2 diabetes (T2D), cardiovascular disease (CVD), and chronic kidney disease [3]. In light of this, NAFLD has been renamed to metabolic dysfunction-associated fatty liver disease (MAFLD) [4], and more recently, metabolic dysfunction-associated steatotic liver disease (MASLD) [5]. Accordingly, steatotic liver disease (SLD) currently consists multiple conditions, including MASLD (characterized by hepatic steatosis along with at least one cardiometabolic risk factor, in the absence of any other identifiable cause), metabolic alcohol-related liver disease (MetALD, patients with MASLD that consumes 140–350 g/wk for females and 210–420 g/wk for males), alcoholic liver disease (ALD, patients that consumes more than 350 g/wk for females and 420 g/wk for males), and etiology-specific/cryptogenic SLD [5]. The proposition of these terms emphasizes the critical role of the liver as a metabolic center. Moreover, the importance of the liver as a central organ in maintaining the metabolic homeostasis is reflected by its interaction with multiple extrahepatic organs, such as the intestines, pancreas, and heart [6, 7]. As such, dysregulation of the metabolic interactions between the liver and other organs may lead to the onset of multiorgan diseases. In this regard, the cross-organ communication between the bone and liver has shown to play an important role in regulating the physiological functions of these organs [8].

Studies in recent years have revealed that nearly all patients with CLD exhibit alterations in bone metabolism, and approximately 75% of CLD patients eventually develop severe osteoporosis [9], indicating a crucial role of liver–bone crosstalk in bone remodeling. To further support the impact of chronic liver diseases on bone metabolism, a recent meta-analysis has shown that patients with NAFLD exhibited a decreased bone mineral density (BMD) and an increased risk of osteoporosis or osteoporotic fractures [10]. However, the studies included in this analysis showed significant heterogeneity, and importantly, it remained unclear how SLD and its different subcategories related to BMD.

In this study, we aimed to clarify the potential association between the commonly proposed subclasses of SLD and bone mass indicators in the general adult population of the United States.

## 2. Materials and Methods

### 2.1. Study Population

**Figure 1:**
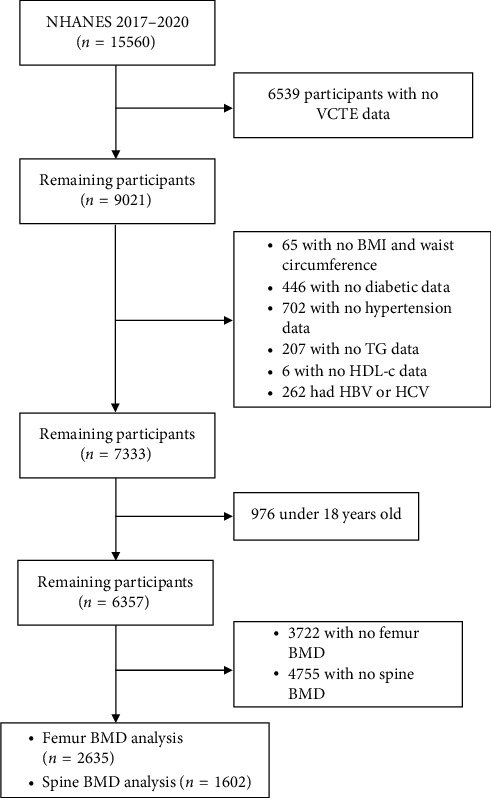
Flowchart of study participants selection. Abbreviations: NHANES: National Health and Nutrition Examination Survey; VCTE: vibration-controlled transient elastography; BMI: body mass index; TG: triglyceride; HDL-c: high-density lipoprotein-cholesterol; HBV: hepatitis B virus; HCV: hepatitis C virus; BMD: bone mineral density.

Data used in this study were obtained from the 2017–2020 cycle of the National Health and Nutrition Examination Survey (NHANES). The following individuals were excluded from the study, including (a) those who were under 18 years old, (b) those who were HBV- and HCV-infected, and (c) those with insufficient or missing data for the assessment of liver stiffness, MASLD, and BMD of the femur and lumbar spine. Based on these exclusion criteria, 2635 and 1602 participants were included for analysis of the femur and lumbar spine, respectively (Figure 1). The protocol for NHANES was approved by the National Center for Health Statistics Research Ethics Review Board, and informed consent was obtained from all participants. Our study received an exemption from the Institutional Review Board because the participants' personal information was fully anonymized.

### 2.2. Assessment of Hepatic Steatosis and Bone Mass

Hepatic steatosis and liver stiffness were quantified using vibration-controlled transient elastography (VCTE) by FibroScan Model 502 V2 Touch machine (Echosens, North America). The machine incorporated controlled attenuation parameter (CAP) to measure ultrasonic attenuation associated with the presence of hepatic steatosis. In the database, hepatic steatosis was defined by a CAP ≥ 288 dB/m. In our analysis, a lower CAP threshold (≥ 248 dB) was used to define hepatic steatosis, as per a published study [11]. CAP and liver stiffness measurement (LSM, kPa) were recorded simultaneously, and the latter was used to identify advanced fibrosis with a cutoff value of ≥ 11.7 kPa [11]. Bone mineral content (BMC, gm), bone area (cm^2^) and BMD (gm/cm^2^) of the femur and spine were measured using dual-energy X-ray absorptiometry (DXA) Hologic QDR 4500 A device (Hologic; Bedford, MA, USA). The femur scans by DXA provide bone density measurements for the total femur, femoral neck, trochanter, intertrochanter, and Ward's triangle. The spine scans by DXA provided bone density measurements for the total spine and vertebrae L1–L4.

### 2.3. Assessment of SLD Subcategories

SLD was defined as the presence of hepatic steatosis, with or without fibrosis. Multiple nomenclatures had been conferred to SLD, including MASLD, MetALD, and ALD. MASLD was defined as a SLD that meets any of the following cardiometabolic risk factors: (1) body mass index (BMI) > 25 kg/m^2^ (23 for Asian participants) or waist circumference > 94 cm (men) or 80 cm (female) or ethnicity adjusted, (2) fasting serum glucose level > 5.6 mmol/L or 2 h postload glucose levels ≥ 7.8 mmol/L or hemoglobin A1c level (HbA1c) ≥ 5.7% or T2D or on treatment for T2D, (3) blood pressure ≥ 130/85 mmHg or on specific antihypertensive drug treatment, (4) plasma triglyceride (TG) level ≥ 1.70 mmol/L or on lipid lowering treatment, and (5) plasma high-density lipoprotein-cholesterol (HDL-c) ≤ 1.0 mmol/L (male) and ≤ 1.3 mmol/L (female) or on lipid-lowering treatment. Other parameters including body weight, height, and blood biochemistry were also obtained. The BMI was calculated as the body weight (kilograms) divided by the body surface area (meter squared) (kg/m^2^). MetALD was defined as daily alcohol intake 20–50 g for females and 30–60 g for males. ALD was defined as excessive alcohol use (> 50 g per day for females and > 60 g per day for males).

### 2.4. Assessment Covariates

Questionnaires containing the sociodemographic information such as age, gender, race or ethnic background, marital status, poverty to income ratio (PIR), education level, serum cotinine, and medical history were used. PIR indicated the socioeconomic status of household participants and estimated household income depending on specific poverty standards for household size by year and state. Covariates of this study included age, gender (male, female), race/ethnicity (Hispanic, non-Hispanic, White, non-Hispanic Black, or other race), marital status (unmarried, married), PIR, and education level (less than 12th grade, high school graduate, some college, and college graduate or above). Serum cotinine was assessed to reflect participants' tobacco exposure. Since the distribution of serum cotinine was highly right-skewed and has low detection rate, it was used as a binary variable (i.e., exposed or unexposed) based on the lower limit of detection (LLOD).

### 2.5. Statistical Analysis

Continuous variables were expressed as mean (standard deviation) and median (max, min), and categorical variables were presented as frequency (percentage). To account for NHANES's complex survey design (e.g., stratification, clustering, and oversampling of specific subgroups), we employed weighted generalized linear models (GLMs) to generate regression coefficients (β) and corresponding 95% confidence intervals (95% CIs), which were used to assess the associations of SLD subcategories, CAP, and LSM with femur and spine BMC, bone area, and BMD. We further utilized weighted generalized additive models (GAMs) to explore potential nonlinear relationships between SLD and BMD. Unlike GLMs, which assume linearity, GAMs flexibly model nonlinear trends via smooth functions, uncovering threshold effects or curvilinear patterns that might otherwise be missed. The unadjusted and fully adjusted models were constructed. Subgroup analysis was performed to assess potential effect modifiers by stratifying sex (male, female), age (< 65, ≥ 65 years), BMI (< 25, ≥ 25 kg/m^2^), glucose status (normal, abnormal), TG (normal, abnormal), HDL-c (normal, abnormal), and LSM (< 11.7, ≥ 11.7 kPa). Abnormal glucose status was defined as fasting serum glucose level > 5.6 mmol/L or confirmed T2D. The abnormal TG was defined as TG level ≥ 1.70 mmol/L or on lipid lowering treatment. The abnormal HDL-c was defined as HDL-c ≤ 1.0 mmol/L (male) or ≤ 1.3 mmol/L (female).

All statistical analyses were conducted using R Version 4.0.5 (Foundation, Vienna, Austria). Statistical significance was defined as a 2-tailed *p* < 0.05.

## 3. Results

### 3.1. General Characteristics of Participants

The general characteristics of the participants with femur and spine data stratified by SLD subcategories are presented in Table 1 and Table S1, respectively. The femur and spine healthy indicators are presented in Table 2 and Table S2, respectively. Compared to normal population, individuals in the SLD subcategories had significant higher BMI, waist circumference, DBP, TG, fasting glucose, LSM, CAP, and femur and spine BMD.

### 3.2. Association Between SLD Subcategories and Bone Mass

Compared to normal participants, MASLD (β = 0.057, 95% CI: 0.038–0.075), MetALD (β = 0.061, 95% CI: 0.016–0.105), and ALD (β = 0.043, 95% CI: 0.007–0.078) exhibited significant positive correlations with total femur BMD (Table 3). MASLD was the only SLD subcategory that was significantly associated with the BMD of all femoral scanning sites. All three SLD subcategories showed a significant association with BMD of trochanter.

For the spine, all three SLD subcategories were significantly associated with BMD of the total spine (MASLD: β = 0.061, 95% CI: 0.027–0.095; MetALD: β = 0.068, 95% CI: 0.031–0.105, ALD: β = 0.070, 95% CI: 0.024–0.116) and vertebrae L1–L4 (Table S3). Of note, a positive association between SLD subcategories and BMC but not the bone area was seen in the femur and spine.

### 3.3. Association of CAP and LSM With Bone Mass

**Figure 2:**
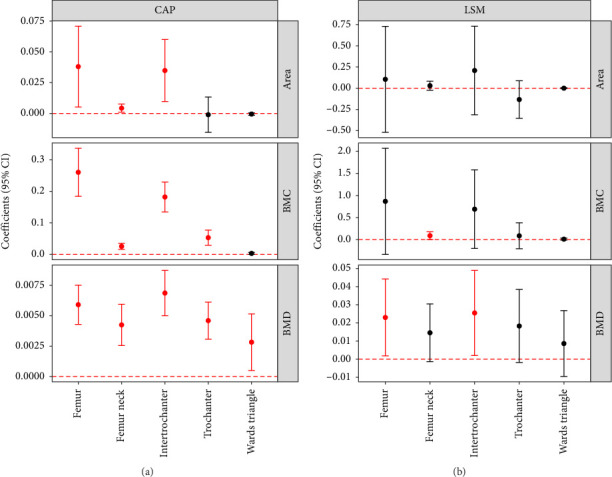
Association of controlled attenuation parameter (CAP) and liver stiffness measurement (LSM) with femur bone mineral density (BMD), bone mineral content (BMC) and bone area.

CAP exhibited a significant positive association with the BMD of the total femur (β = 0.006, 95% CI: 0.004–0.008) and that of all femoral scanning sites (Figure 2). CAP was also associated with the bone area and BMC of some femoral scanning sites (total femur, femoral neck, and intertrochanter). LSM was only associated with BMD of the total femur (β = 0.023, 95% CI: 0.002–0.044) and intertrochanter (β = 0.025, 95% CI: 0.002–0.049).

For the spine, CAP was significantly associated with BMD and BMC of the total spine and vertebrae L1–L4 (Figure S1). LSM also exhibited a significant positive association with BMD of the total spine (β = 0.037, 95% CI: 0.007–0.066) and vertebrae L1–L4.

**Figure 3:**
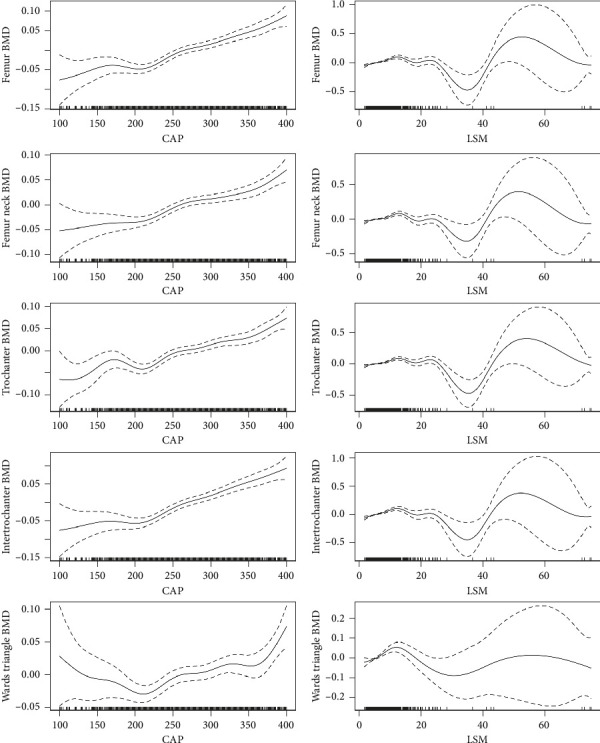
Exposure-response relationship of controlled attenuation parameter (CAP) and liver stiffness measurement (LSM) with femur bone mineral density (BMD).

Overall, a trend of a positive correlation between CAP and various bone healthy indicators (Figure 3, Figure S16) as well as a nonlinear association between LSM and various bone healthy indicators were observed in our study.

### 3.4. Subgroup Analysis

Among participants with LSM < 11.7 kPa, SLD subcategories exhibited a significantly stronger positive association with femur and spine BMD (*p* < 0.05) (Tables S4–S17).

A significant association between CAP and femur BMD was only observed among participants with CAP > 248 dB/m and LSM < 11.7 kPa (*p* < 0.05) (Figures S2–S8). The positive association between LSM and femur BMD was significantly stronger among female participants and those with LSM < 11.7 kPa (*p* < 0.05), whereas a negative association between LSM and femur BMD existed among participants with LSM ≥ 11.7 kPa (*p* < 0.05).

The positive association between LSM and spine BMD was significantly stronger among female participants and those with abnormal HDL and LSM < 11.7 kPa (*p* < 0.05) (Figures S9–S15). The significant association between CAP and spine BMD was only observed among participants with CAP > 248 dB/m and LSM < 11.7 kPa (*p* < 0.05), whereas the significant association between LSM and spine BMD was only observed among participants with age < 65 years, BMI ≥ 25 kg/m^2^, CAP > 248 dB/m, and LSM < 11.7 kPa (*p* < 0.05).

## 4. Discussion

Fatty liver disease has emerged as the most common type of CLD globally, posing a major health burden [11]. NAFLD represents a most common variant of fatty liver disease. With the deep understanding of the pathogenesis of NAFLD, this disease entity has been renamed MAFLD [4], and more recently a new term “MASLD” was proposed [5, 12]. The naming shift from the previous terms is based on the assumption that previous terms used exclusive criteria to affirm the diagnosis, oversighted considerable proportion of lean individuals with normal BMI, and certain metabolic risk factors (such as HOMA-IR and hsCRP) were infrequently used in routine clinical practice. The newly named MASLD affects approximately 30% of individuals worldwide and has become the dominant contributor to CLD, with a substantial economic burden exceeding $100 billion in the United States alone [13, 14].

The recent research has revealed that liver–bone crosstalk plays an essential role in bone metabolism. To evaluate if the new term MASLD faithfully reflects its systemic metabolic impact, a cross-sectional study was conducted to evaluate the relationship between SLD subcategories and various skeletal health indicators (BMC, bone area, and BMD) of the femur and spine since the establishment of the MASLD definition. Our results indicated that, after adjusting for covariates, all three SLD subgroups (MASLD, MetALD, and ALD) were significantly associated with increased BMD and BMC of the femur and spine but not with the bone area in these regions.

Existing evidence regarding the association between fatty liver disease and BMD remained inconclusive. A cross-sectional analysis based on the NHANES 2017-2018 data involving 2031 participants (1070 men and 961 women) aged 50 years and above revealed an association between MAFLD, liver stiffness, and higher femoral and lumbar BMD although significance diminished upon adjustment for BMI [15]. In a similar study [16], MAFLD patients aged 50 years and older were found to have a higher femoral BMD and a lower risk of osteoporosis compared to those without MAFLD, which was in line with our findings. However, Ciardullo et al. utilized the same database and individuals of the same age group and found no correlation between NAFLD, fibrosis, and femoral neck BMD [17]. Moreover, among individuals aged 20 to 59 years, an independent adverse relationship was observed between NAFLD and lumbar BMD [18]. Whereas our subgroup analysis revealed that among individuals aged < 65 years, MASLD was positively correlated with BMD and BMC at all femoral scan sites (the total femur, femoral neck, trochanter, intertrochanter, and Ward's triangle), as well as at all spine scan sites (the total spine and vertebrae L1–L4). Specifically, MASLD was positively correlated only with L3 BMD. MetALD exhibited positive correlations solely with BMD in the trochanter and all spine scan sites. ALD demonstrated positive correlations with BMD in the total femur, trochanter, and all spine scan sites. In contrast, among individuals aged ≥ 65 years, MASLD demonstrated positive correlations with BMD in all femoral scan sites and all spine scan sites except for L2. MetALD was positively correlated with BMD in all femoral scan sites and all spine scan sites except for L4. Interestingly, ALD showed a negative correlation only with L2 bone area.

Thus, MASLD, MAFLD, and NAFLD may identify diverse populations with varying bone metabolism. Such a difference may reflect the difference in the diagnostic criteria used for these terms. Our study represented the first study using the NHANES 2017–2020 cycle data to analyze the association between BMD and SLD in a larger sample size. In the present study, we found that MASLD, MetALD, and ALD were all significantly associated with increased femoral and spinal BMD. Specifically, among individuals with abnormal glucose and LSM < 11.7 kPa, MASLD displayed a stronger positive correlation with femoral and spinal BMD. In females with normal HDL and LSM < 11.7 kPa, MetALD exhibited a significantly positive association with femoral and spinal BMD. Similarly, in female participants younger than 65 years with abnormal HDL and LSM < 11.7 kPa, ALD demonstrated a markedly stronger positive correlation with femoral and spinal BMD.

CAP, which was obtained simultaneously with LSM, quantified hepatic steatosis by measuring the ultrasonic attenuation of echo waves [19] and had shown to accurately measure the level of fat infiltration independent of other clinical factors such as obesity and liver enzyme levels [20]. A weighted GAM was employed to evaluate the relationships between CAP, LSM, and BMD, in order to reflect the associations between hepatic steatosis and fibrosis with BMD. We observed that in individuals with the CAP > 248 dB/m, there existed a positive relationship between CAP and BMD at nearly all femoral and spinal sites. Conversely, the relationship between LSM and BMD was nonlinear.

It is noteworthy that studies on the association between the severity of hepatic steatosis and BMD were limited, and the results were controversial. Previous studies had identified an obesity paradox between children and adults: Obese children and adolescents were more prone to fractures, whereas obesity appeared to have a protective effect against fractures in adults [21]. In a study based on the NHANES 2017–2018 data, NAFLD adults with CAP < 302 dB/m were negatively associated with lumbar BMD [18]. However, this association disappeared after adjusting for covariates, in that the individuals with CAP ≥ 302 dB/m did not show any significant correlation between severe steatosis and BMD. In other studies, a negative correlation between hepatic fat content and BMD was observed in middle-aged and elderly Chinese men [22] and in children from Spain and San Diego [23, 24].

In our study, a positive association between CAP and femoral and spinal BMD was identified only in participants with CAP > 248 dB/m, BMI ≥ 25 kg/m^2^, and LSM < 11.7 kPa. This was consistent with the results of a longitudinal cohort study involving 4536 individuals and 13,354 person-years of total follow-up of 2.1 years, which demonstrated that NAFLD had a significantly protective effect on BMD in women compared to non-NAFLD [25]. Our study showed that during the progression from steatosis to fibrosis, MASLD appeared to be protective against BMD, possibly due to the beneficial effects of the higher mechanical loading for bone health as a result of higher body weight [26–28]. In vivo and in vitro studies have demonstrated that reduced mechanical loading increases osteocyte apoptosis. Mechanically stimulated osteocytes not only attenuated osteocyte apoptosis through the Caveolin-1/ERK and Wnt/β-catenin pathways [29–31] but also partially activated the NO pathway to secrete soluble factors that inhibited osteoclast formation and bone resorption [32, 33], thereby contributing to a positive bone balance. Another possible reason was that the degree of hepatic steatosis in our population is relatively mild, which might explain the observed positive correlation. Moreover, it should be considered that increased body fat percentage in obese individuals may also interfere with BMD estimates from DXA scans [25]. The mechanistic link between liver steatosis and bone metabolism still requires further validation through in vivo and in vitro studies.

Our study also revealed a significantly heightened positive correlation between LSM and BMD in female participants with LSM < 11.7 kPa, whereas in individuals with LSM ≥ 11.7 kPa, LSM demonstrated a negative correlation with femoral BMD. Our findings underscored the dichotomous role of fatty liver on bone metabolism. On one hand, in female patients, fatty liver exerted a protective effect on BMD before progressing to fibrosis, and this was consistent with the published data indicating that overweight and obesity were beneficial to BMD in women [26–28]. Such beneficial role of fat on bone health in women was attributable to the regulatory role of sex hormones, particularly estrogen on bone mass [34]. Extensive research indicated that estrogen played a crucial role in bone metabolism [35, 36]. Researchers selectively deleted estrogen receptor (ER) alpha in the monocyte/macrophage lineage of mice and observed an extended lifespan of osteoclasts, which accelerated trabecular bone loss [37]. Mechanically, estrogen modulated osteoclast formation and activity through complex interactions with the TNF-α, TGF-β, and OPG/RANKL/RANK systems, ultimately suppressing bone resorption [38]. Compared to the general population, obese women had higher serum estrogen levels, primarily due to estrogen's various effects in the brain, adipose tissue, and other peripheral sites [39]. Specifically, estrogen not only increased food intake and reduces energy expenditure but also promoted the quantity and regional redistribution of adipose tissue, leading to specific pathophysiological changes in adipose tissue. Moreover, the Women's Health Initiative Observational Study concluded that the higher the levels of bioavailable estradiol and testosterone in postmenopausal women, the lower the risk of osteoporotic fractures [40]. On the other hand, the adverse effects of fatty liver on BMD onset when fibrosis develops, regardless of gender, as demonstrated in a recent study [41] that both mineral bone density and microstructure in obese populations progressively deteriorated with increasing FIB-4 levels, independent of classic risk factors for metabolic disorders such as impaired glucose tolerance and T2D. Kim et al. also observed similar outcomes in their study [42]. Besides, Zhang et al. revealed that hepatic fibrosis index (FIB-4 score) was negatively associated with BMD T-scores in T2D patients [43]. The declined protective effect of fat on bone mass during the progression of liver fibrosis may be attributed to multiple factors, which still need further elucidation through in vivo and in vitro experiments.

We utilized a recently released expanded population to analyze the relationship between the latest designated SLD subgroups and various bone health markers in the femur and spine. However, several limitations of our study should be noted. First, this study is limited to the data from the 2017–2020 cycle of NHANES, and the study subjects included U.S. population, more validation studies are warranted to generalize our findings to the entire hepatology community. Second, the cross-sectional design of this study means that a temporal and causal relationship between SLD subgroups and BMD is lacking. Third, our study relied VCTE for identifying fatty liver and the assessment of the degree of hepatic steatosis. Although VCTE has been widely used as a well-established approach for diagnosing fatty liver [44–46], liver biopsy is by far the gold standard for confirming fatty liver disease.

In conclusion, a positive correlation was identified between SLD and bone metabolic status; however, this association may be influenced by liver fibrosis. Our findings may contribute to a deeper understanding of the liver–bone axis interaction, offering insights into the prevention and treatment strategies for hepatic osteodystrophy. Accordingly, studies using large-scale and long-term follow-up cohorts are warranted to further elucidate the relationship between the two, particularly with regard to gender and racial disparities.

## Figures and Tables

**Table 1:** Summary of participants and femur data stratified by SLD subcategories.

Variable	Normal (*n* = 1612)	MASLD (*n* = 774)	MetALD (*n* = 172)	ALD (*n* = 77)	Total (*n* = 2635)	*p*
Gender						< 0.001
Male	791 (49.1%)	458 (59.2%)	83 (48.3%)	57 (74.0%)	1389 (52.7%)	
Female	821 (50.9%)	316 (40.8%)	89 (51.7%)	20 (26.0%)	1246 (47.3%)	
Age (years)						< 0.001
Mean (SD)	64.4 (9.10)	64.5 (8.74)	60.9 (7.07)	58.8 (6.61)	64.0 (8.90)	
Median [min, max]	63.0 [50.0, 80.0]	64.0 [50.0, 80.0]	60.0 [50.0, 80.0]	57.0 [50.0, 80.0]	63.0 [50.0, 80.0]	
Race						< 0.001
Hispanic	295 (18.3%)	174 (22.5%)	48 (27.9%)	37 (48.1%)	554 (21.0%)	
Non-Hispanic White	617 (38.3%)	320 (41.3%)	69 (40.1%)	27 (35.1%)	1033 (39.2%)	
Non-Hispanic Black	438 (27.2%)	157 (20.3%)	38 (22.1%)	9 (11.7%)	642 (24.4%)	
Other race	262 (16.3%)	123 (15.9%)	17 (9.9%)	4 (5.2%)	406 (15.4%)	
Cotinine						0.016
Yes	934 (57.9%)	428 (55.3%)	99 (57.6%)	57 (74.0%)	1518 (57.6%)	
No	678 (42.1%)	346 (44.7%)	73 (42.4%)	20 (26.0%)	1117 (42.4%)	
Season						0.158
Cold	811 (50.3%)	393 (50.8%)	94 (54.7%)	48 (62.3%)	1346 (51.1%)	
Warm	801 (49.7%)	381 (49.2%)	78 (45.3%)	29 (37.7%)	1289 (48.9%)	
Education level						0.026
Less than 12th grade	291 (18.1%)	131 (16.9%)	30 (17.4%)	17 (22.1%)	469 (17.8%)	
High school graduate	382 (23.7%)	183 (23.6%)	43 (25.0%)	26 (33.8%)	634 (24.1%)	
Some college	481 (29.8%)	260 (33.6%)	58 (33.7%)	27 (35.1%)	826 (31.3%)	
College graduate or above	458 (28.4%)	200 (25.8%)	41 (23.8%)	7 (9.1%)	706 (26.8%)	
Marital status						< 0.001
Married	939 (58.3%)	523 (67.6%)	110 (64.0%)	50 (64.9%)	1622 (61.6%)	
Unmarried	673 (41.7%)	251 (32.4%)	62 (36.0%)	27 (35.1%)	1013 (38.4%)	
PIR						0.596
Mean (SD)	2.87 (1.63)	2.82 (1.59)	3.00 (1.68)	2.76 (1.77)	2.86 (1.63)	
Median [min, max]	2.59 [0, 5.00]	2.49 [0.0300, 5.00]	3.05 [0, 5.00]	2.47 [0, 5.00]	2.59 [0, 5.00]	
BMI (kg/m^2^)						< 0.001
Mean (SD)	27.2 (5.00)	31.9 (6.03)	32.5 (6.04)	33.3 (6.09)	29.1 (5.93)	
Median [min, max]	26.6 [15.7, 53.3]	30.9 [18.3, 56.3]	31.4 [22.4, 58.7]	32.5 [22.7, 52.8]	28.3 [15.7, 58.7]	
Waist (cm)						< 0.001
Mean (SD)	96.2 (12.1)	109 (13.3)	109 (12.2)	113 (13.4)	101 (14.0)	
Median [min, max]	95.9 [65.0, 145]	108 [70.3, 164]	108 [80.2, 149]	112 [83.3, 148]	100 [65.0, 164]	
SBP (mmHg)						0.651
Mean (SD)	131 (19.5)	131 (18.1)	132 (20.0)	133 (21.2)	131 (19.2)	
Median [min, max]	128 [82.7, 214]	130 [78.7, 204]	130 [92.7, 210]	129 [90.3, 187]	129 [78.7, 214]	
DBP (mmHg)						< 0.001
Mean (SD)	74.1 (11.0)	75.4 (11.4)	78.1 (11.3)	79.6 (11.1)	74.9 (11.2)	
Median [min, max]	73.3 [41.3, 121]	75.3 [41.3, 117]	76.0 [56.3, 122]	78.0 [49.0, 107]	74.3 [41.3, 122]	
HDL-C (mmol/L)						< 0.001
Mean (SD)	1.51 (0.437)	1.25 (0.329)	1.37 (0.381)	1.35 (0.424)	1.42 (0.420)	
Median [min, max]	1.45 [0.130, 4.84]	1.19 [0.570, 2.64]	1.29 [0.650, 2.51]	1.24 [0.750, 2.77]	1.34 [0.130, 4.84]	
TG (mmol/L)						< 0.001
Mean (SD)	1.44 (1.23)	1.96 (1.27)	2.07 (1.98)	1.87 (1.05)	1.64 (1.32)	
Median [min, max]	1.20 [0.418, 33.0]	1.59 [0.418, 14.3]	1.66 [0.508, 17.3]	1.61 [0.677, 6.20]	1.34 [0.418, 33.0]	
Fasting glucose (mmol/L)						< 0.001
Mean (SD)	6.11 (1.92)	7.32 (2.86)	6.48 (1.77)	6.45 (1.81)	6.49 (2.28)	
Median [min, max]	5.66 [3.22, 25.0]	6.38 [2.66, 22.2]	6.08 [4.05, 16.3]	6.00 [4.16, 12.2]	5.83 [2.66, 25.0]	
LSM (kPa)						< 0.001
Mean (SD)	5.42 (4.79)	6.71 (4.49)	6.00 (2.94)	7.46 (5.70)	5.90 (4.67)	
Median [min, max]	4.80 [2.10, 75.0]	5.70 [1.60, 75.0]	5.10 [2.30, 25.3]	6.00 [2.50, 43.5]	5.00 [1.60, 75.0]	
CAP (dB/m)						< 0.001
Mean (SD)	235 (36.9)	331 (32.2)	328 (29.7)	337 (31.3)	272 (58.6)	
Median [min, max]	240 [100, 287]	324 [288, 400]	324 [288, 400]	331 [288, 400]	271 [100, 400]	

*Note:* MASLD: metabolic dysfunction-associated steatotic liver disease; MetALD: metabolic alcohol-related liver disease; PIR: poverty to income ratio; TG: triglyceride.

Abbreviations: ALD, alcoholic liver disease; BMI, body mass index; CAP, controlled attenuation parameter; DBP, diastolic blood pressure; HDL-c, high-density lipoprotein-cholesterol; LSM, liver stiffness measurement; SBP, systolic blood pressure.

**Table 2:** Femur healthy indicators stratified by SLD subcategories.

Variable	Normal	MASLD	MetALD	ALD	Total	*p*
	(*n* = 1612)	(*n* = 774)	(*n* = 172)	(*n* = 77)	(*n* = 2635)	
Femur BMD						< 0.001
Mean (SD)	0.908 (0.157)	0.971 (0.156)	0.976 (0.149)	1.02 (0.118)	0.934 (0.159)	
Median [min, max]	0.898 [0.432, 1.44]	0.971 [0.552, 1.61]	0.974 [0.551, 1.46]	1.01 [0.677, 1.37]	0.926 [0.432, 1.61]	
Femur BMC						< 0.001
Mean (SD)	34.4 (9.65)	37.9 (10.3)	36.9 (9.40)	41.3 (8.66)	35.8 (9.96)	
Median [min, max]	33.2 [13.5, 66.7]	37.0 [17.0, 91.5]	35.8 [19.5, 59.9]	40.9 [22.4, 68.5]	34.8 [13.5, 91.5]	
Femur area						< 0.001
Mean (SD)	37.6 (6.42)	38.6 (6.35)	37.4 (5.78)	40.3 (6.02)	37.9 (6.38)	
Median [min, max]	36.7 [23.6, 60.2]	38.2 [23.2, 61.6]	35.9 [28.4, 57.0]	40.4 [26.6, 53.3]	37.2 [23.2, 61.6]	
Femur neck BMD						< 0.001
Mean (SD)	0.756 (0.145)	0.795 (0.149)	0.810 (0.142)	0.845 (0.0999)	0.774 (0.147)	
Median [min, max]	0.742 [0.355, 1.42]	0.781 [0.435, 1.70]	0.809 [0.402, 1.24]	0.850 [0.608, 1.09]	0.762 [0.355, 1.70]	
Femur neck BMC						< 0.001
Mean (SD)	3.95 (0.933)	4.22 (0.986)	4.24 (0.909)	4.55 (0.713)	4.07 (0.954)	
Median [min, max]	3.85 [0.140, 8.46]	4.15 [2.17, 10.8]	4.16 [2.19, 6.99]	4.45 [3.00, 6.79]	3.97 [0.140, 10.8]	
Femur neck area						0.003
Mean (SD)	5.21 (0.589)	5.29 (0.565)	5.22 (0.519)	5.38 (0.521)	5.24 (0.577)	
Median [min, max]	5.20 [0.310, 6.96]	5.28 [3.39, 7.23]	5.21 [3.72, 6.45]	5.37 [4.13, 6.34]	5.23 [0.310, 7.23]	
Trochanter BMD						< 0.001
Mean (SD)	0.681 (0.128)	0.732 (0.130)	0.737 (0.120)	0.774 (0.108)	0.702 (0.130)	
Median [min, max]	0.673 [0, 1.09]	0.722 [0.413, 1.26]	0.729 [0.362, 1.22]	0.774 [0.492, 1.04]	0.695 [0, 1.26]	
Trochanter BMC						< 0.001
Mean (SD)	8.20 (2.46)	9.01 (2.59)	8.73 (2.29)	9.53 (2.18)	8.51 (2.51)	
Median [min, max]	7.86 [0, 21.4]	8.71 [3.24, 19.6]	8.48 [4.12, 15.9]	9.84 [4.52, 14.2]	8.18 [0, 21.4]	
Trochanter area						0.026
Mean (SD)	11.9 (2.33)	12.2 (2.17)	11.8 (1.99)	12.3 (1.96)	12.0 (2.26)	
Median [min, max]	11.7 [0, 25.1]	12.0 [6.60, 21.8]	11.4 [7.30, 17.4]	12.4 [8.06, 16.8]	11.8 [0, 25.1]	
Intertrochanter BMD						< 0.001
Mean (SD)	1.08 (0.187)	1.16 (0.183)	1.16 (0.176)	1.20 (0.142)	1.11 (0.188)	
Median [min, max]	1.07 [0.488, 1.68]	1.16 [0.618, 1.81]	1.16 [0.707, 1.64]	1.18 [0.784, 1.63]	1.11 [0.488, 1.81]	
Intertrochanter BMC						< 0.001
Mean (SD)	22.3 (6.66)	24.6 (7.11)	23.9 (6.62)	27.2 (6.26)	23.2 (6.90)	
Median [min, max]	21.4 [8.14, 43.1]	23.9 [7.79, 61.2]	22.8 [11.2, 42.4]	27.1 [14.9, 47.5]	22.5 [7.79, 61.2]	
Intertrochanter area						< 0.001
Mean (SD)	20.4 (4.16)	21.1 (4.25)	20.4 (3.90)	22.7 (4.11)	20.7 (4.19)	
Median [min, max]	19.9 [11.4, 36.7]	20.8 [7.69, 35.0]	19.7 [14.3, 36.1]	22.9 [13.9, 31.0]	20.2 [7.69, 36.7]	
Wards triangle BMD						< 0.001
Mean (SD)	0.554 (0.161)	0.582 (0.166)	0.607 (0.167)	0.623 (0.131)	0.568 (0.163)	
Median [min, max]	0.535 [0.141, 1.34]	0.570 [0.196, 1.54]	0.585 [0.264, 1.08]	0.610 [0.405, 1.10]	0.549 [0.141, 1.54]	
Wards triangle BMC						< 0.001
Mean (SD)	0.652 (0.209)	0.684 (0.209)	0.723 (0.225)	0.732 (0.169)	0.669 (0.210)	
Median [min, max]	0.620 [0.0600, 1.72]	0.660 [0.210, 1.72]	0.690 [0.280, 1.40]	0.720 [0.430, 1.42]	0.640 [0.0600, 1.72]	
Wards triangle area						0.312
Mean (SD)	1.17 (0.0870)	1.17 (0.0818)	1.19 (0.0871)	1.17 (0.0772)	1.17 (0.0853)	
Median [min, max]	1.16 [0.230, 1.32]	1.16 [0.990, 1.32]	1.16 [1.04, 1.32]	1.16 [1.03, 1.32]	1.16 [0.230, 1.32]	

*Note:* MASLD: metabolic dysfunction-associated steatotic liver disease; MetALD: metabolic alcohol-related liver disease.

Abbreviations: ALD, alcoholic liver disease; BMC, bone mineral content; BMD, bone mineral density.

**Table 3:** Association of SLD subcategories with femur BMD, BMC, and bone area.

		Normal	MASLD	MetALD	ALD
			β (95% CI)	β (95% CI)	β (95% CI)
Total femur	BMD	Reference	**0.057 (0.038, 0.075)**	**0.061 (0.016, 0.105)**	**0.043 (0.007, 0.078)**
	BMC	Reference	**2.247 (1.251, 3.243)**	**1.930 (0.039, 3.821)**	1.764 (−0.250, 3.778)
	Area	Reference	0.058 (−0.427, 0.542)	−0.217 (−0.911, 0.477)	0.289 (−1.093, 1.671)

Femur neck	BMD	Reference	**0.035 (0.015, 0.056)**	**0.045 (0.003, 0.086)**	0.030 (−0.010, 0.071)
	BMC	Reference	**0.207 (0.084, 0.330)**	0.247 (−0.002, 0.496)	0.147 (−0.070, 0.363)
	Area	Reference	0.028 (−0.023, 0.078)	0.034 (−0.050, 0.118)	0.005 (−0.146, 0.157)

Trochanter	BMD	Reference	**0.042 (0.024, 0.061)**	**0.051 (0.016, 0.086)**	**0.050 (0.019, 0.080)**
	BMC	Reference	**0.421 (0.131, 0.710)**	**0.535 (0.075, 0.994)**	0.290 (−0.320, 0.900)
	Area	Reference	−0.122 (−0.363, 0.120)	−0.056 (−0.299, 0.186)	−0.373 (−0.927, 0.180)

Intertrochanter	BMD	Reference	**0.069 (0.049, 0.089)**	**0.071 (0.019, 0.124)**	0.033 (−0.012, 0.078)
	BMC	Reference	**1.619 (0.953, 2.286)**	1.150 (−0.166, 2.465)	**1.327 (0.023, 2.632)**
	Area	Reference	0.153 (−0.190, 0.495)	−0.194 (−0.765, 0.377)	0.659 (−0.149, 1.467)

Wards triangle	BMD	Reference	**0.034 (0.012, 0.057)**	0.017 (−0.042, 0.076)	0.010 (−0.040, 0.059)
	BMC	Reference	**0.041 (0.013, 0.069)**	0.016 (−0.063, 0.095)	0.005 (−0.058, 0.068)
	Area	Reference	0.002 (−0.009, 0.014)	−0.010 (−0.033, 0.013)	−0.007 (−0.030, 0.016)

*Note:* Boldface type indicates statistical significance. MASLD: metabolic dysfunction-associated steatotic liver disease; MetALD: metabolic alcohol-related liver disease.

Abbreviations: ALD, alcoholic liver disease; BMC, bone mineral content; BMD, bone mineral density.

## Data Availability

The data that support the findings of this study are available from the corresponding authors upon reasonable request.

## Nomenclature

MASLD: Metabolic dysfunction-associated steatotic liver disease
NAFLD: Nonalcoholic fatty liver disease
MAFLD: Dysfunction-associated fatty liver disease
SLD: Steatotic liver disease
NHANES: National Health and Nutrition Examination Survey
VCTE: Vibration-controlled transient elastography
DXA: Dual-energy X-ray absorptiometry
BMC: Bone mineral content
BMD: Bone mineral density
CAP: Controlled attenuation parameter
LSM: Liver stiffness measurement
MetALD: Metabolic alcohol-related liver disease
ALD: Alcoholic liver disease
T2D: Type 2 diabetes
CVD: Cardiovascular disease
CLD: Chronic liver disease
BMI: Body mass index
HbA1c: Hemoglobin A1c level
TG: Triglyceride
HDL-c: High-density lipoprotein-cholesterol
HBV: Hepatitis B virus
HCV: Hepatitis C virus
PIR: Poverty to income ratio
LLOD: Lower limit of detection
ER: Estrogen receptor

## Supporting Information

Supporting Information 1 Supporting Figure 1: Association of CAP and LSM with spine BMD, BMC, and bone area.

Supporting Information 2 Supporting Figure 2: Association of CAP and LSM with femur BMD, BMC, and bone area stratified by age.

Supporting Information 3 Supporting Figure 3: Association of CAP and LSM with femur BMD, BMC, and bone area stratified by gender.

Supporting Information 4 Supporting Figure 4: Association of CAP and LSM with femur BMD, BMC, and bone area stratified by BMI.

Supporting Information 5 Supporting Figure 5: Association of CAP and LSM with femur BMD, BMC, and bone area stratified by glucose status.

Supporting Information 6 Supporting Figure 6: Association of CAP and LSM with femur BMD, BMC, and bone area stratified by the TG status.

Supporting Information 7 Supporting Figure 7: Association of CAP and LSM with femur BMD, BMC, and bone area stratified by HDL-c status.

Supporting Information 8 Supporting Figure 8: Association of CAP and LSM with femur BMD, BMC, and bone area stratified by the LSM value.

Supporting Information 9 Supporting Figure 9: Association of CAP and LSM with spine BMD, BMC, and bone area stratified by age.

Supporting Information 10 Supporting Figure 10: Association of CAP and LSM with spine BMD, BMC, and bone area stratified by gender.

Supporting Information 11 Supporting Figure 11: Association of CAP and LSM with spine BMD, BMC, and bone area stratified by BMI.

Supporting Information 12 Supporting Figure 12: Association of CAP and LSM with spine BMD, BMC, and bone area stratified by glucose status.

Supporting Information 13 Supporting Figure 13: Association of CAP and LSM with spine BMD, BMC, and bone area stratified by TG status.

Supporting Information 14 Supporting Figure 14: Association of CAP and LSM with spine BMD, BMC, and bone area stratified by HDL-c status.

Supporting Information 15 Supporting Figure 15: Association of CAP and LSM with spine BMD, BMC, and bone area stratified by LSM value.

Supporting Information 16 Supporting Figure 16: Exposure–response relationship of CAP and LSM with spine BMD.

Supporting Information 17 Supporting Table 1: Summary of participants with spine data stratified by SLD subcategories. Supporting Table 2: Summary of the spine healthy indicators stratified by SLD subcategories. Supporting Table 3: Association of SLD subcategories with spine BMD, BMC, and bone area. Supporting Table 4: Association of SLD subcategories with femur BMD, BMC, and bone area stratified by age. Supporting Table 5: Association of SLD subcategories with femur BMD, BMC, and bone area stratified by sex. Supporting Table 6: Association of SLD subcategories with femur BMD, BMC, and bone area stratified by BMI. Supporting Table 7: Association of SLD subcategories with femur BMD, BMC, and bone area stratified by glucose status. Supporting Table 8: Association of SLD subcategories with femur BMD, BMC, and bone area stratified by TG. Supporting Table 9: Association of SLD subcategories with femur BMD, BMC, and bone area stratified by HDL. Supporting Table 10: Association of SLD subcategories with femur BMD, BMC, and bone area stratified by LSM. Supporting Table 11: Association of SLD subcategories with spine BMD, BMC, and bone area stratified by age. Supporting Table 12: Association of SLD subcategories with spine BMD, BMC, and bone area stratified by sex. Supporting Table 13: Association of SLD subcategories with spine BMD, BMC, and bone area stratified by BMI. Supporting Table 14: Association of SLD subcategories with spine BMD, BMC, and bone area stratified by glucose status. Supporting Table 15: Association of SLD subcategories with spine BMD, BMC, and bone area stratified by TG. Supporting Table 16: Association of SLD subcategories with spine BMD, BMC, and bone area stratified by HDL. Supporting Table 17: Association of SLD subcategories with spine BMD, BMC, and bone area stratified by LSM.
