# Supplementary figures and images for: Increased BMD in SLD Patients Without Advanced Hepatic Fibrosis: Evidence From the NHANES 2017–2020 Database
Source: Can J Gastroenterol Hepatol. 2025 Aug 11;2025:6969761. doi: 10.1155/cjgh/6969761 (PMC12360881; doi:10.1155/cjgh/6969761)

## Slide 1
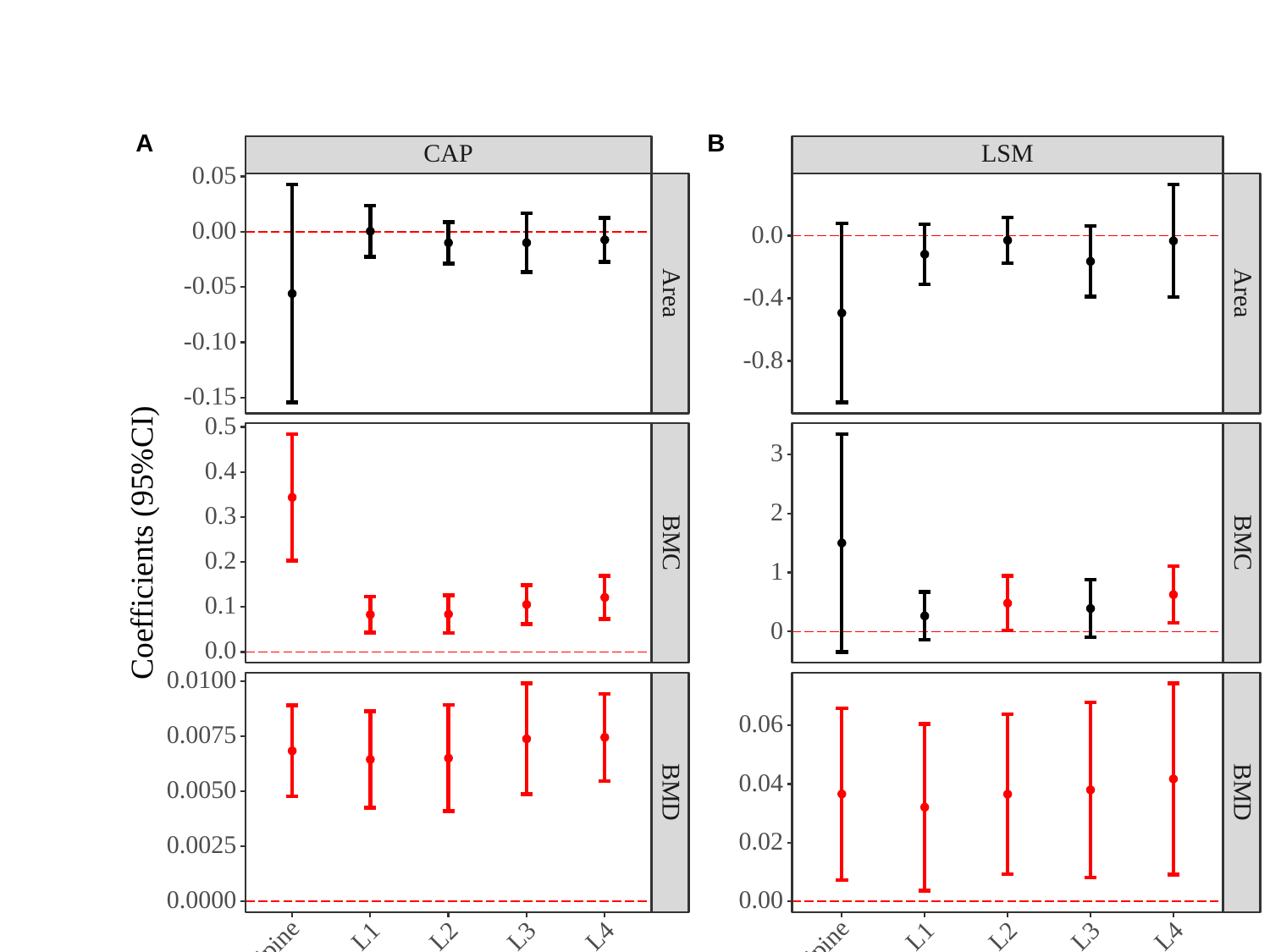

A
B
CAP
LSM
0.05
0.00
0.0
-0.05
Area
Area
-0.4
-0.10
-0.8
-0.15
0.5
3
0.4
2
0.3
Coefficients (95%CI)
BMC
BMC
0.2
1
0.1
0
0.0
0.0100
0.06
0.0075
0.04
0.0050
BMD
BMD
0.02
0.0025
0.0000
0.00
L3
L3
L1
L2
L4
L1
L2
L4
Spine
Spine

Supplement: Supporting Information 1 — Supporting Figure 1: Association of CAP and LSM with spine BMD, BMC, and bone area. [file 6969761.f1.pptx]

## Slide 1
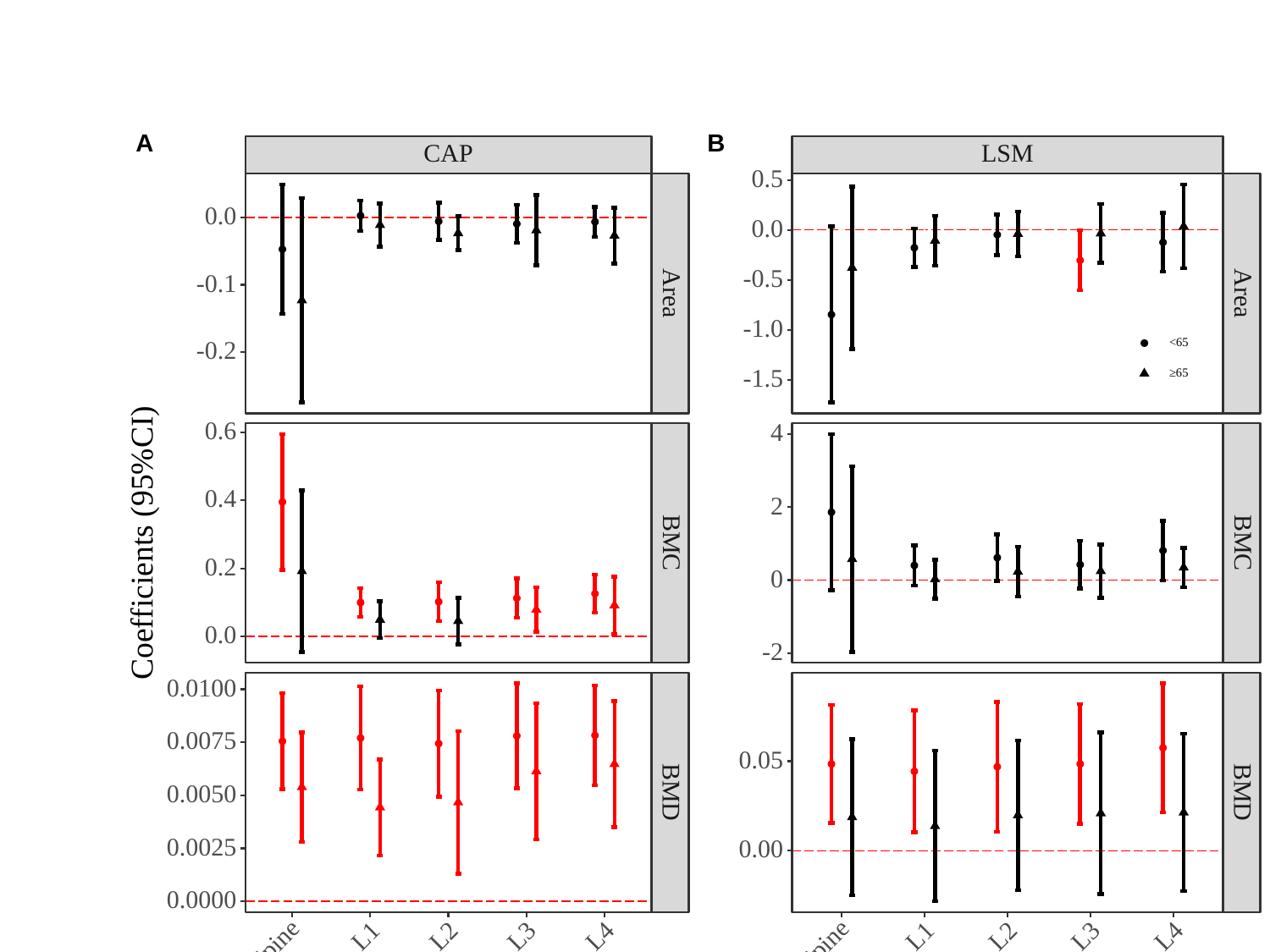

A
B
CAP
LSM
0.5
*
*
*
*
*
*
*
0.0
*
*
*
*
*
*
*
*
0.0
*
*
*
*
*
-0.5
-0.1
Area
Area
-1.0
<65
-0.2
≥65
-1.5
0.6
4
*
*
*
0.4
*
2
*
Coefficients (95%CI)
BMC
BMC
*
*
*
*
*
*
0.2
*
0
*
*
*
*
*
*
*
*
0.0
-2
0.0100
*
*
*
*
*
*
*
*
*
*
*
*
0.0075
*
*
*
*
*
*
0.05
*
*
BMD
BMD
0.0050
0.0025
0.00
0.0000
L3
L3
L1
L2
L4
L1
L2
L4
Spine
Spine

Supplement: Supporting Information 9 — Supporting Figure 9: Association of CAP and LSM with spine BMD, BMC, and bone area stratified by age. [file 6969761.f9.pptx]

## Slide 1
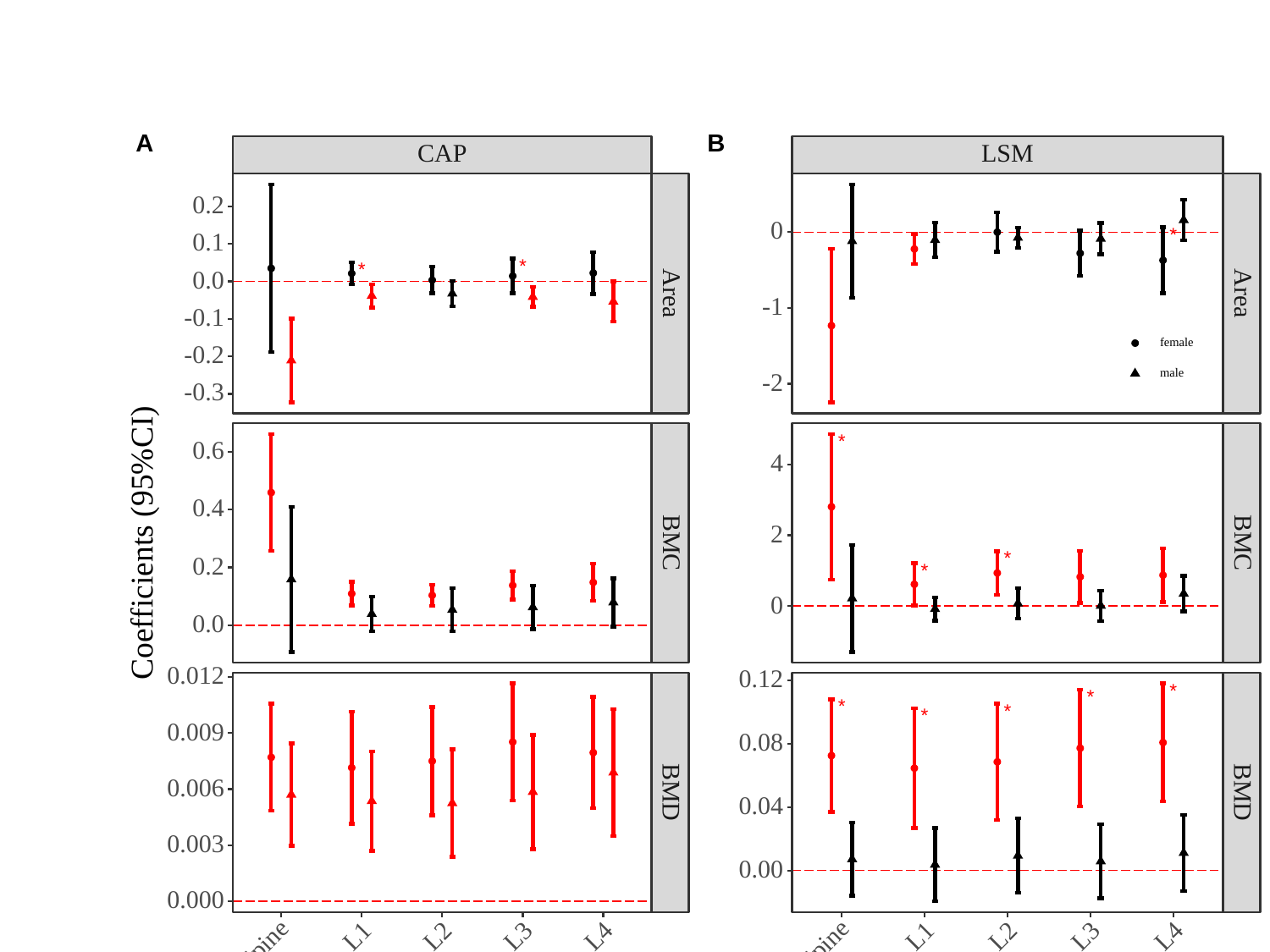

A
B
CAP
LSM
*
*
0.2
*
*
0
*
*
*
*
0.1
*
*
*
*
*
*
0.0
*
Area
Area
*
*
*
*
-1
-0.1
*
female
-0.2
male
-2
-0.3
*
*
0.6
4
0.4
*
2
Coefficients (95%CI)
BMC
BMC
*
*
*
*
0.2
*
*
*
*
*
*
*
*
*
*
*
0
*
*
0.0
0.012
0.12
*
*
*
*
*
*
*
*
*
*
*
0.009
0.08
*
*
*
*
0.006
BMD
BMD
0.04
*
*
*
*
*
0.003
0.00
0.000
L3
L3
L1
L2
L4
L1
L2
L4
Spine
Spine

Supplement: Supporting Information 10 — Supporting Figure 10: Association of CAP and LSM with spine BMD, BMC, and bone area stratified by gender. [file 6969761.f10.pptx]

## Slide 1
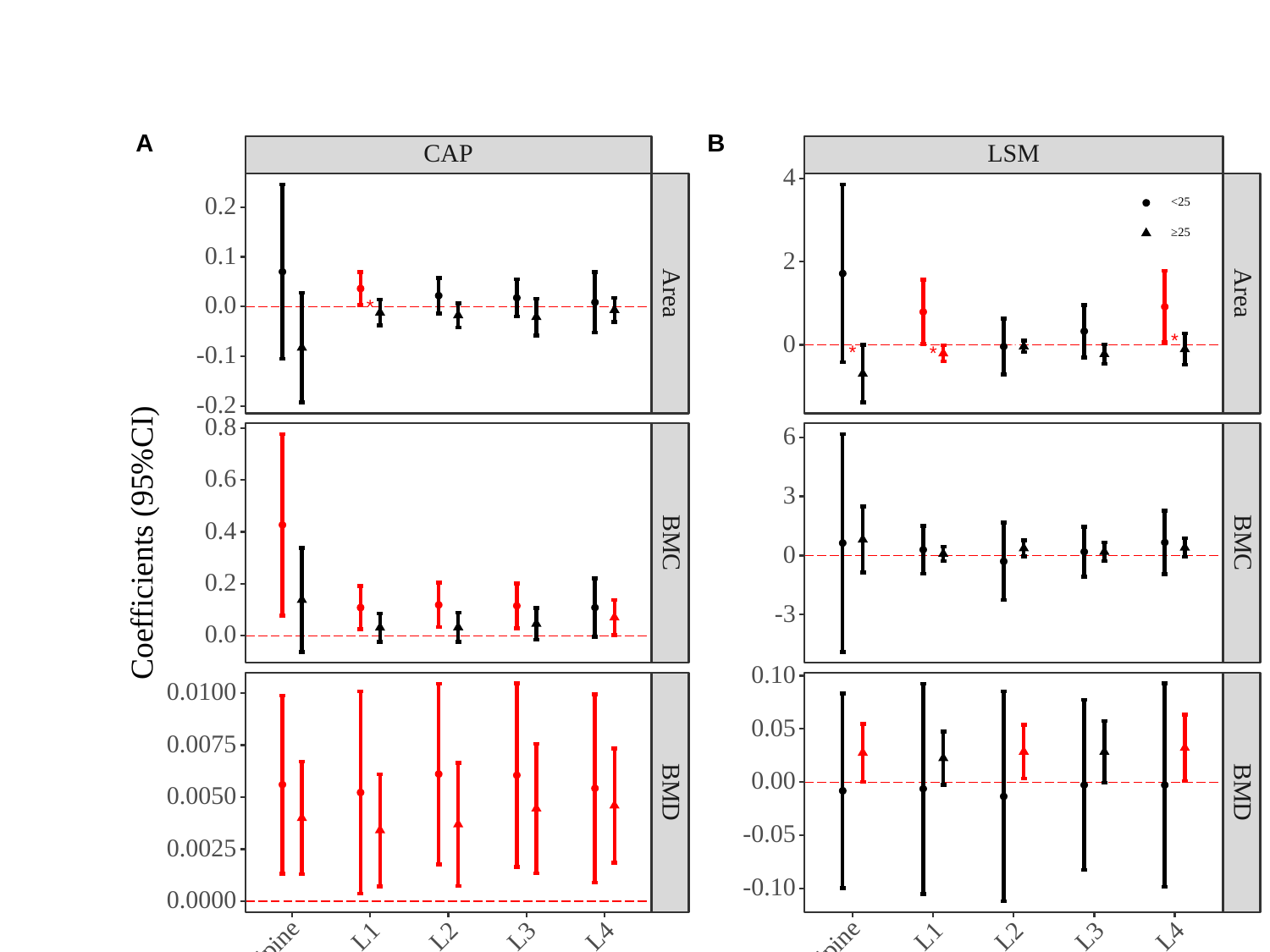

A
B
CAP
LSM
4
*
*
0.2
<25
≥25
0.1
2
*
*
*
*
Area
Area
*
*
0.0
*
*
*
*
*
*
*
0
*
*
-0.1
*
*
*
-0.2
0.8
6
*
*
0.6
3
*
*
0.4
*
Coefficients (95%CI)
*
*
BMC
BMC
*
*
0
*
*
*
0.2
*
*
*
*
*
-3
*
*
*
0.0
0.10
0.0100
*
*
*
*
*
*
*
*
*
*
0.05
*
*
*
*
0.0075
*
*
*
*
*
0.00
*
BMD
BMD
0.0050
-0.05
0.0025
-0.10
0.0000
L3
L3
L1
L2
L4
L1
L2
L4
Spine
Spine

Supplement: Supporting Information 11 — Supporting Figure 11: Association of CAP and LSM with spine BMD, BMC, and bone area stratified by BMI. [file 6969761.f11.pptx]

## Slide 1
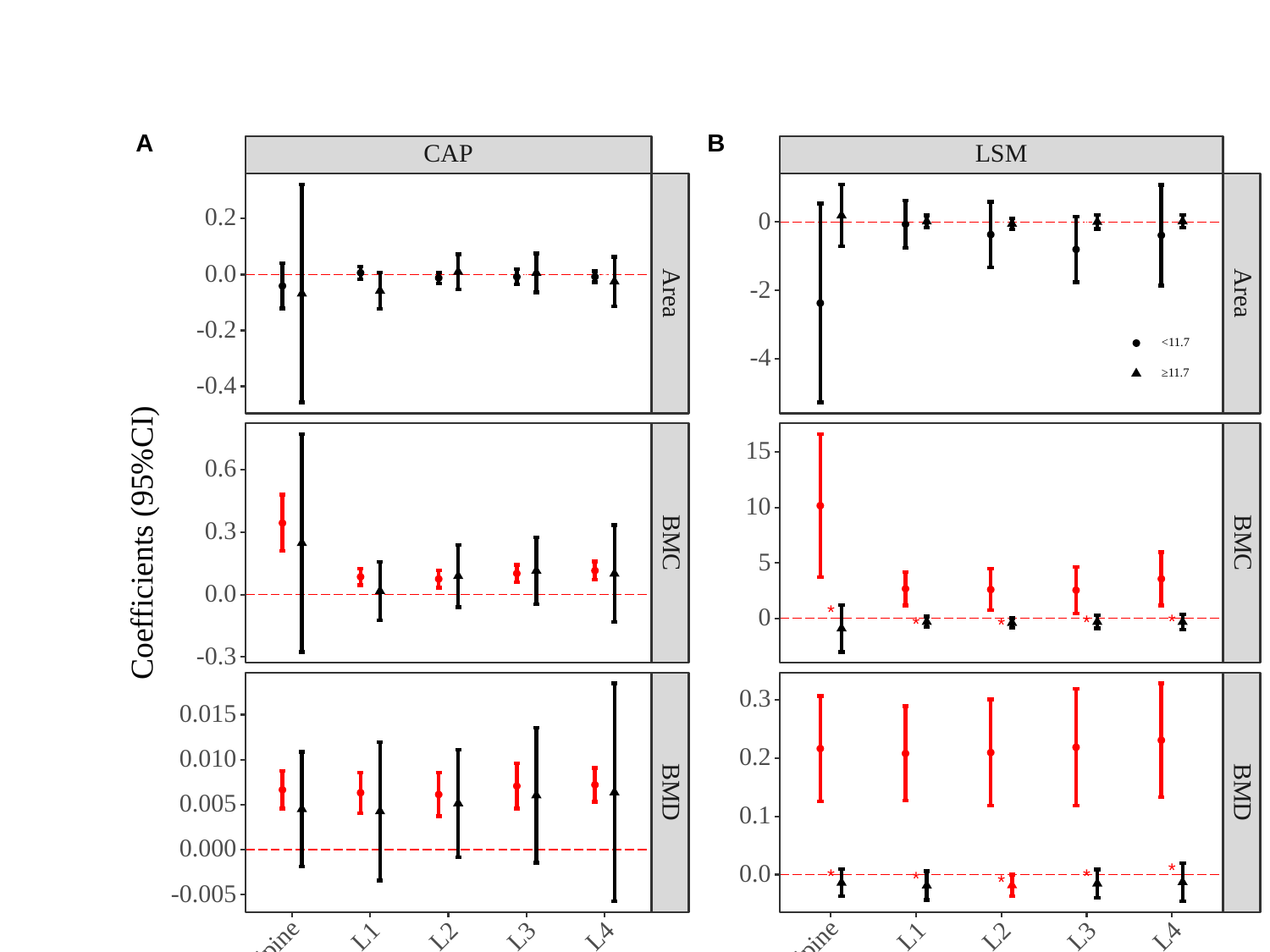

A
B
CAP
LSM
*
*
*
*
*
*
0.2
0
*
*
*
*
*
*
*
*
0.0
*
*
*
*
*
*
-2
Area
Area
-0.2
<11.7
-4
≥11.7
-0.4
*
*
15
0.6
10
*
0.3
Coefficients (95%CI)
*
BMC
BMC
*
*
5
*
*
*
*
*
*
*
*
*
0.0
0
*
*
*
*
*
-0.3
*
*
0.3
*
*
*
0.015
*
*
*
0.2
0.010
*
*
*
*
*
*
*
BMD
BMD
0.005
0.1
0.000
0.0
*
*
*
*
*
-0.005
L3
L3
L1
L2
L4
L1
L2
L4
Spine
Spine

Supplement: Supporting Information 15 — Supporting Figure 15: Association of CAP and LSM with spine BMD, BMC, and bone area stratified by LSM value. [file 6969761.f15.pptx]
