# Supplementary material for: Increased BMD in SLD Patients Without Advanced Hepatic Fibrosis: Evidence From the NHANES 2017–2020 Database
Source: Can J Gastroenterol Hepatol. 2025 Aug 11;2025:6969761. doi: 10.1155/cjgh/6969761 (PMC12360881; doi:10.1155/cjgh/6969761)
Supplement: Supporting Information 2 — Supporting Figure 2: Association of CAP and LSM with femur BMD, BMC, and bone area stratified by age. [file 6969761.f2.pptx]

## Slide 1
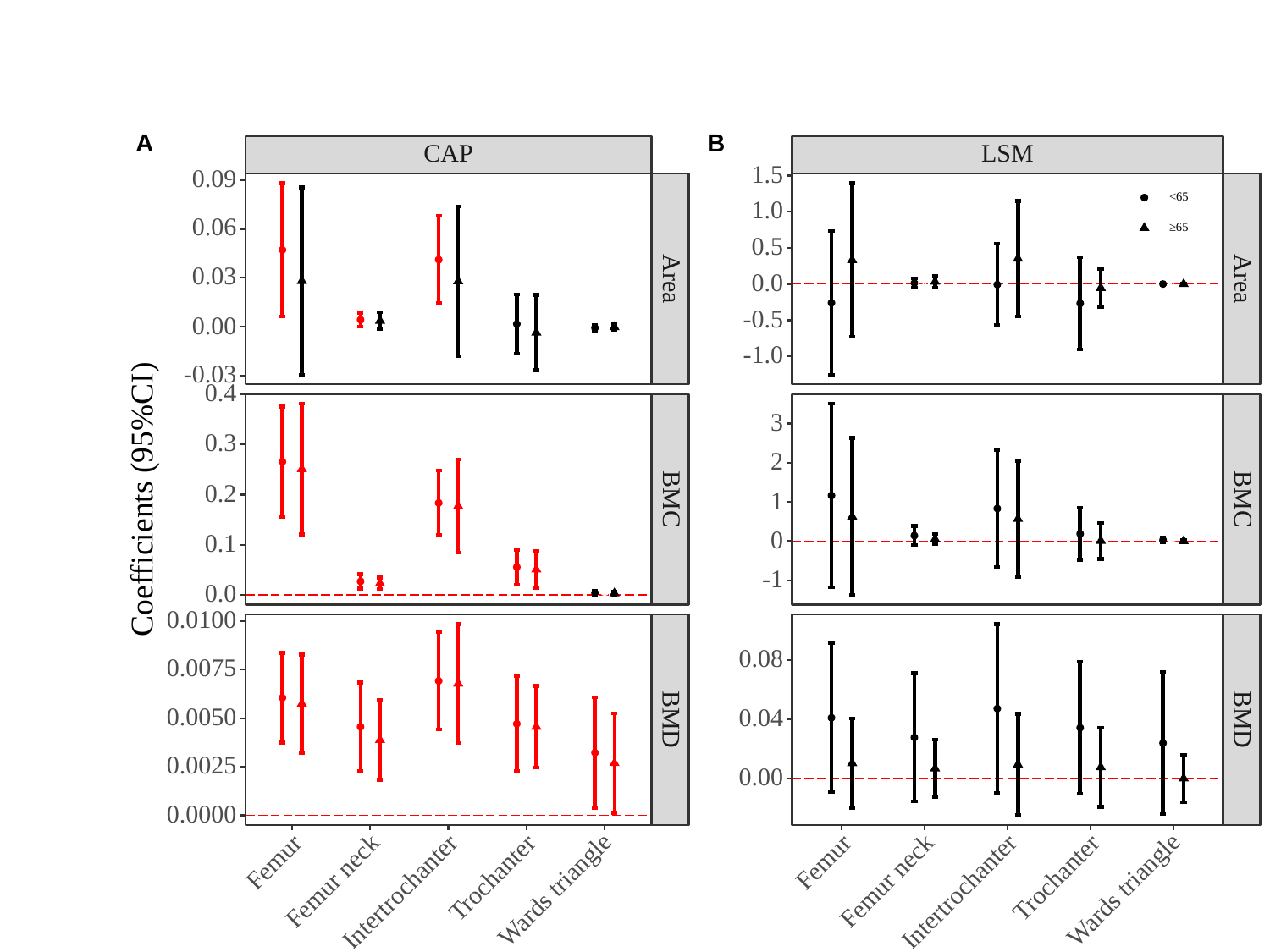

A
B
CAP
LSM
1.5
0.09
*
*
*
<65
1.0
*
*
0.06
*
≥65
*
0.5
*
*
0.03
Area
Area
*
0.0
*
*
*
*
*
*
-0.5
*
0.00
*
*
*
-1.0
-0.03
0.4
*
*
*
3
0.3
*
2
*
*
*
*
Coefficients (95%CI)
0.2
BMC
BMC
1
*
*
*
0
0.1
*
*
*
*
*
-1
*
*
0.0
*
*
0.0100
*
*
*
*
0.08
*
*
0.0075
*
*
*
*
*
*
*
*
0.0050
0.04
BMD
BMD
*
*
*
*
*
0.0025
*
0.00
0.0000
Femur
Femur
Trochanter
Trochanter
Femur neck
Femur neck
Wards triangle
Wards triangle
Intertrochanter
Intertrochanter
