# Supplementary material for: Increased BMD in SLD Patients Without Advanced Hepatic Fibrosis: Evidence From the NHANES 2017–2020 Database
Source: Can J Gastroenterol Hepatol. 2025 Aug 11;2025:6969761. doi: 10.1155/cjgh/6969761 (PMC12360881; doi:10.1155/cjgh/6969761)
Supplement: Supporting Information 3 — Supporting Figure 3: Association of CAP and LSM with femur BMD, BMC, and bone area stratified by gender. [file 6969761.f3.pptx]

## Slide 1
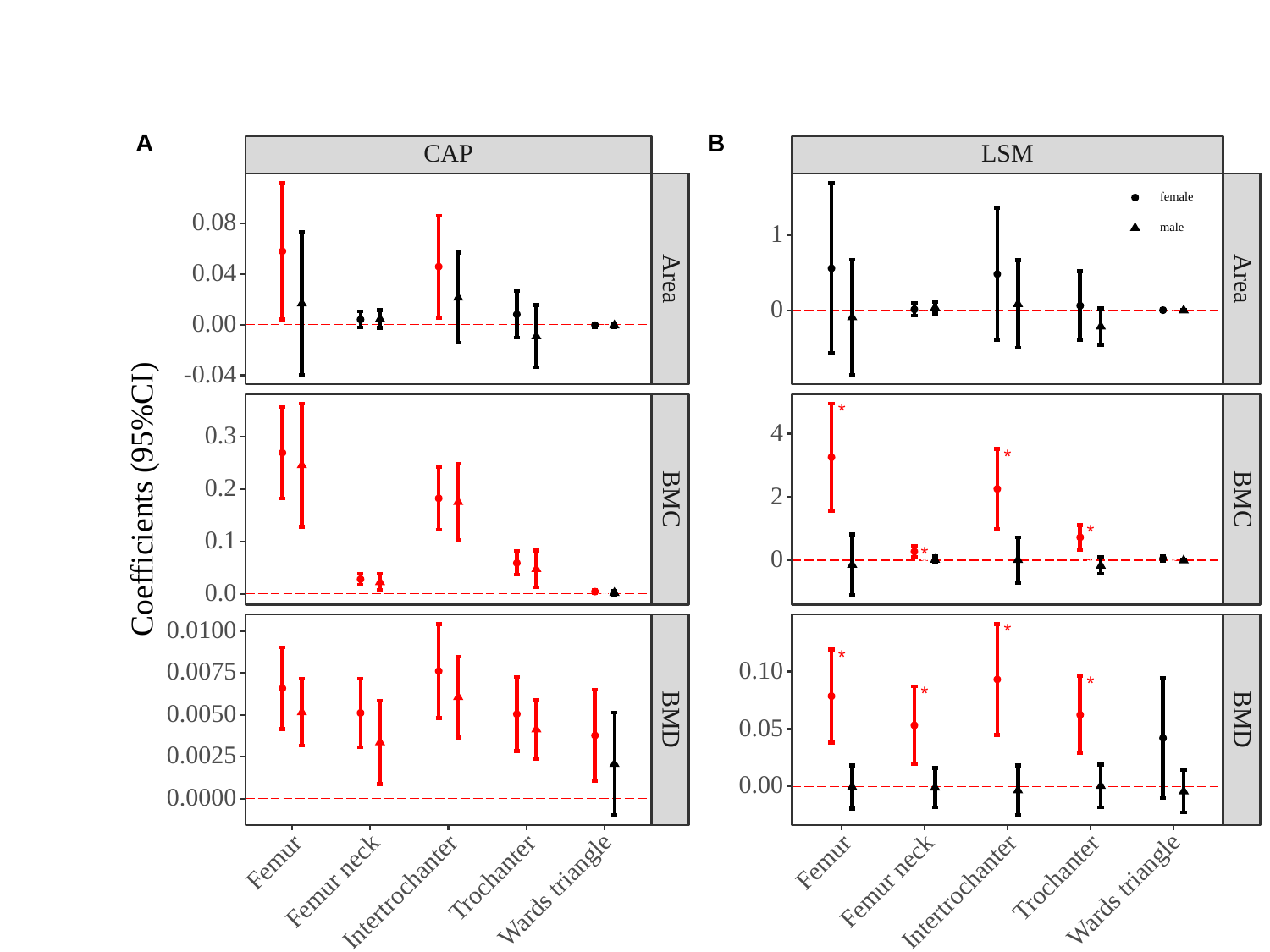

A
B
CAP
LSM
*
*
female
*
0.08
*
male
1
*
*
0.04
*
*
Area
Area
*
*
0
*
*
*
*
*
*
0.00
*
*
*
*
-0.04
*
*
*
4
0.3
*
*
*
0.2
Coefficients (95%CI)
2
BMC
BMC
*
0.1
*
*
0
*
*
*
*
*
*
*
*
*
0.0
*
*
0.0100
*
*
*
*
*
0.10
0.0075
*
*
*
*
*
*
*
*
0.0050
*
BMD
BMD
*
0.05
0.0025
*
*
*
*
*
0.00
0.0000
Femur
Femur
Trochanter
Trochanter
Femur neck
Femur neck
Wards triangle
Wards triangle
Intertrochanter
Intertrochanter
