# Supplementary material for: Increased BMD in SLD Patients Without Advanced Hepatic Fibrosis: Evidence From the NHANES 2017–2020 Database
Source: Can J Gastroenterol Hepatol. 2025 Aug 11;2025:6969761. doi: 10.1155/cjgh/6969761 (PMC12360881; doi:10.1155/cjgh/6969761)
Supplement: Supporting Information 4 — Supporting Figure 4: Association of CAP and LSM with femur BMD, BMC, and bone area stratified by BMI. [file 6969761.f4.pptx]

## Slide 1
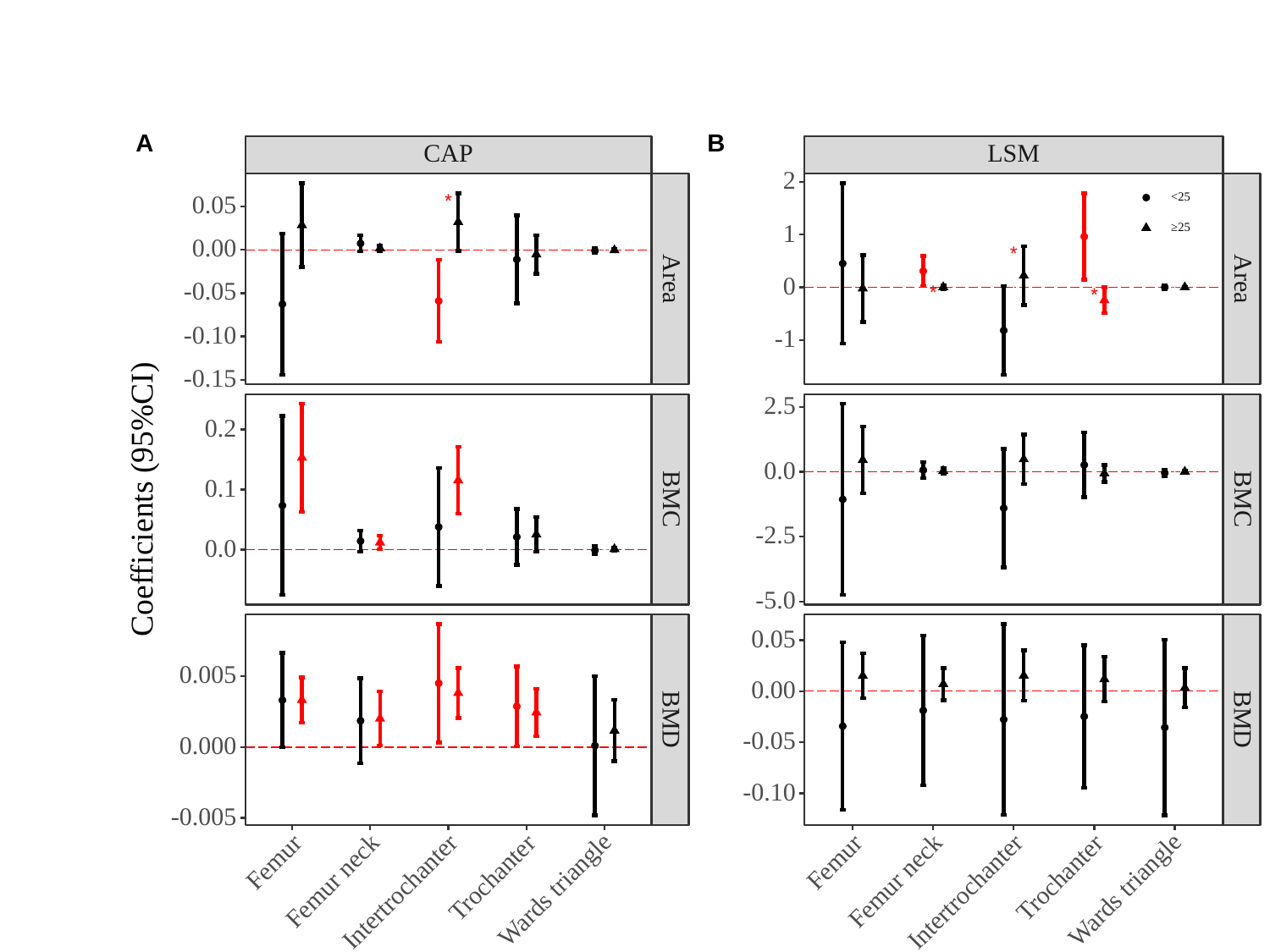

A
B
CAP
LSM
2
*
*
<25
0.05
*
*
*
≥25
1
*
*
*
0.00
*
*
*
*
*
*
*
Area
Area
0
-0.05
*
*
*
*
*
-0.10
-1
-0.15
2.5
*
*
0.2
*
*
*
*
*
*
0.0
*
*
*
*
*
*
0.1
Coefficients (95%CI)
BMC
BMC
*
*
-2.5
*
0.0
*
*
*
-5.0
*
*
0.05
*
*
*
*
*
*
*
*
0.005
*
*
*
*
*
0.00
*
*
*
*
*
BMD
BMD
-0.05
0.000
-0.10
-0.005
Femur
Femur
Trochanter
Trochanter
Femur neck
Femur neck
Wards triangle
Wards triangle
Intertrochanter
Intertrochanter
