# Supplementary material for: Increased BMD in SLD Patients Without Advanced Hepatic Fibrosis: Evidence From the NHANES 2017–2020 Database
Source: Can J Gastroenterol Hepatol. 2025 Aug 11;2025:6969761. doi: 10.1155/cjgh/6969761 (PMC12360881; doi:10.1155/cjgh/6969761)
Supplement: Supporting Information 5 — Supporting Figure 5: Association of CAP and LSM with femur BMD, BMC, and bone area stratified by glucose status. [file 6969761.f5.pptx]

## Slide 1
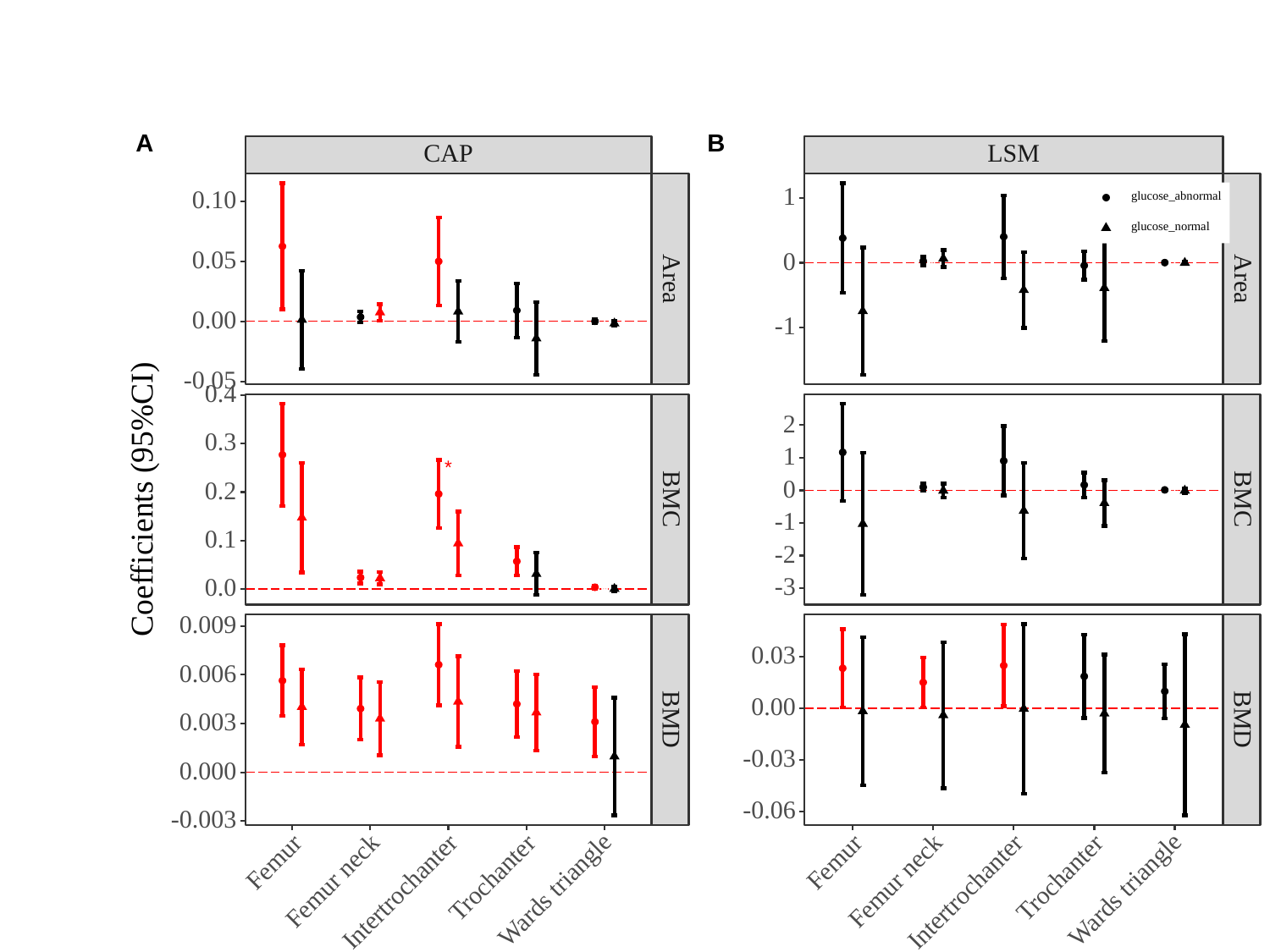

A
B
CAP
LSM
*
*
1
glucose_abnormal
0.10
*
glucose_normal
*
*
0.05
*
0
*
*
*
*
*
*
Area
Area
*
*
*
*
*
0.00
*
-1
*
*
-0.05
0.4
*
*
2
*
0.3
1
*
*
*
*
*
0
0.2
Coefficients (95%CI)
*
*
*
BMC
BMC
*
*
-1
*
0.1
-2
*
*
*
*
-3
0.0
*
*
0.009
*
*
*
*
*
*
*
0.03
*
*
*
*
*
0.006
*
*
*
*
*
*
*
0.00
*
BMD
BMD
0.003
-0.03
0.000
-0.06
-0.003
Femur
Femur
Trochanter
Trochanter
Femur neck
Femur neck
Wards triangle
Wards triangle
Intertrochanter
Intertrochanter
