# Supplementary material for: Increased BMD in SLD Patients Without Advanced Hepatic Fibrosis: Evidence From the NHANES 2017–2020 Database
Source: Can J Gastroenterol Hepatol. 2025 Aug 11;2025:6969761. doi: 10.1155/cjgh/6969761 (PMC12360881; doi:10.1155/cjgh/6969761)
Supplement: Supporting Information 7 — Supporting Figure 7: Association of CAP and LSM with femur BMD, BMC, and bone area stratified by HDL-c status. [file 6969761.f7.pptx]

## Slide 1
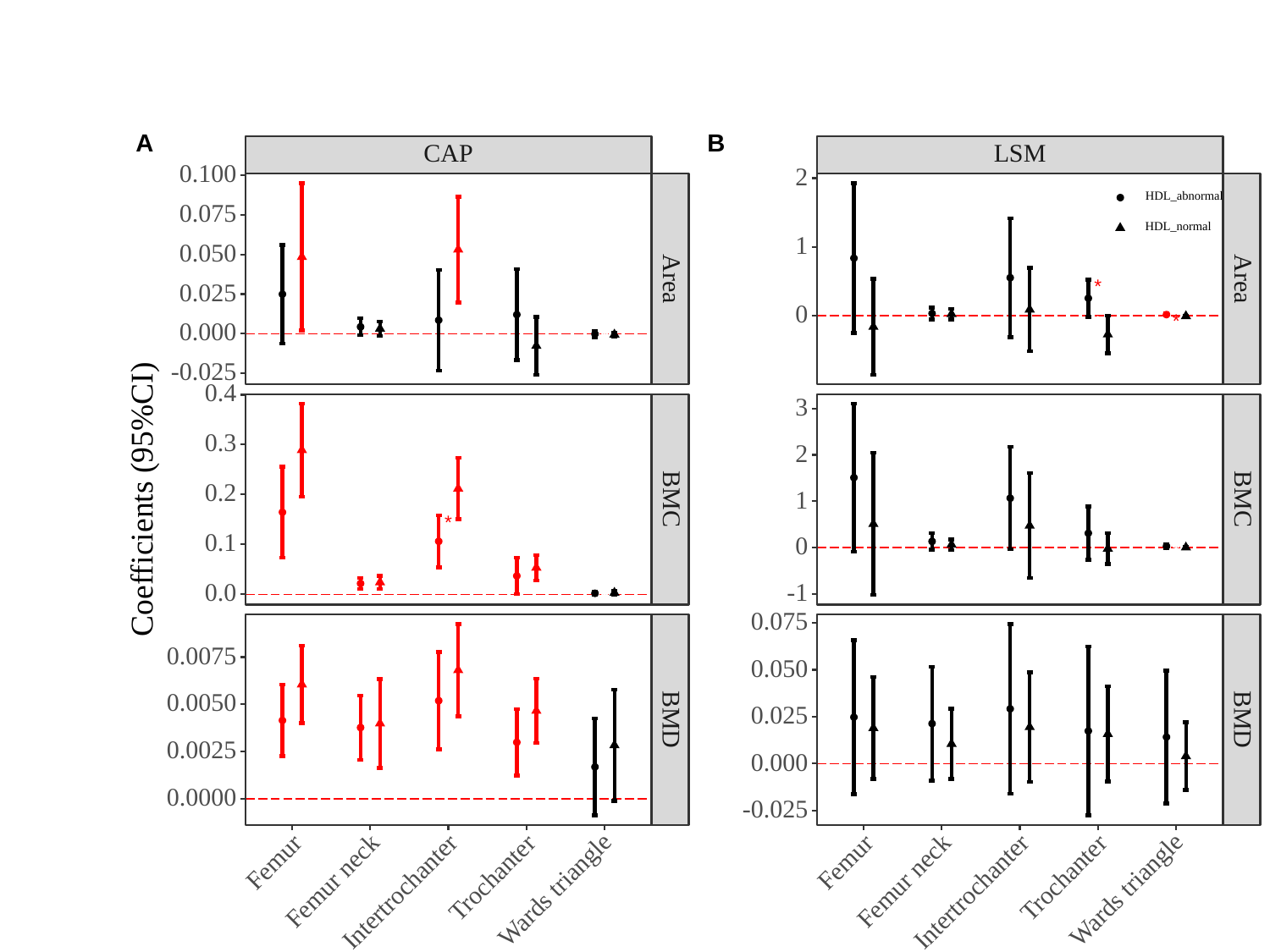

A
B
CAP
LSM
0.100
2
*
*
HDL_abnormal
*
0.075
HDL_normal
*
1
0.050
*
Area
Area
*
*
*
*
0.025
*
0
*
*
*
*
*
*
*
0.000
*
*
*
-0.025
0.4
3
*
*
0.3
2
*
*
*
*
*
Coefficients (95%CI)
0.2
BMC
BMC
1
*
*
0.1
0
*
*
*
*
*
*
*
*
*
-1
0.0
*
*
0.075
*
*
*
0.0075
*
*
*
0.050
*
*
*
*
*
*
*
*
*
0.0050
*
0.025
BMD
BMD
*
*
*
*
0.0025
0.000
0.0000
-0.025
Femur
Femur
Trochanter
Trochanter
Femur neck
Femur neck
Wards triangle
Wards triangle
Intertrochanter
Intertrochanter
