# Supplementary material for: Increased BMD in SLD Patients Without Advanced Hepatic Fibrosis: Evidence From the NHANES 2017–2020 Database
Source: Can J Gastroenterol Hepatol. 2025 Aug 11;2025:6969761. doi: 10.1155/cjgh/6969761 (PMC12360881; doi:10.1155/cjgh/6969761)
Supplement: Supporting Information 8 — Supporting Figure 8: Association of CAP and LSM with femur BMD, BMC, and bone area stratified by the LSM value. [file 6969761.f8.pptx]

## Slide 1
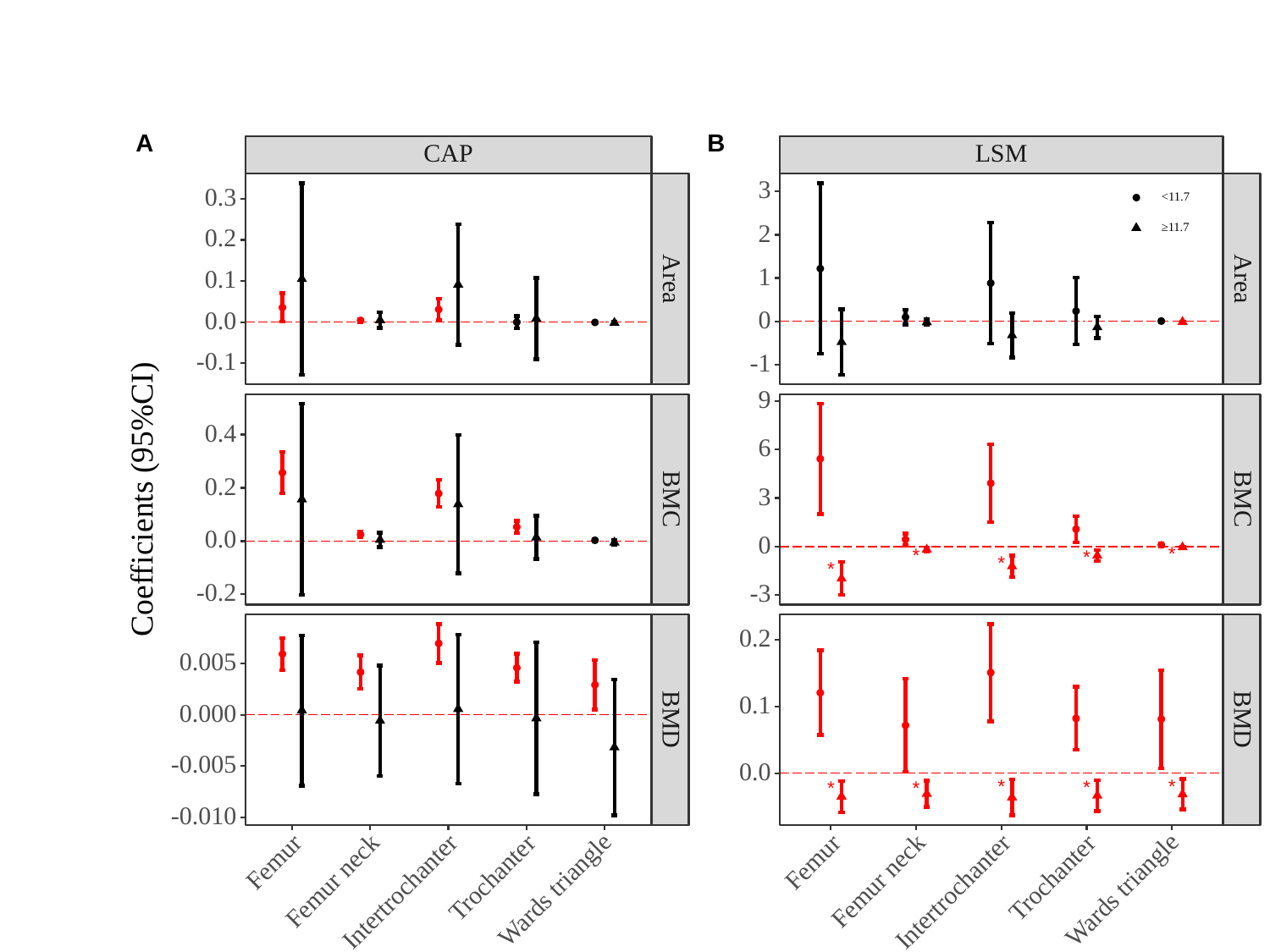

A
B
CAP
LSM
3
*
*
0.3
<11.7
≥11.7
2
*
*
0.2
1
Area
Area
0.1
*
*
*
*
0
0.0
*
*
*
*
*
*
*
*
*
*
*
*
-0.1
-1
9
*
*
0.4
6
*
*
*
0.2
Coefficients (95%CI)
*
3
BMC
BMC
*
*
*
0.0
*
0
*
*
*
*
*
*
*
*
*
*
-0.2
-3
*
*
0.2
*
*
*
*
0.005
*
*
*
*
*
*
*
*
*
0.1
0.000
BMD
BMD
-0.005
0.0
*
*
*
*
*
-0.010
Femur
Femur
Trochanter
Trochanter
Femur neck
Femur neck
Wards triangle
Wards triangle
Intertrochanter
Intertrochanter
