# Supplementary material for: Increased BMD in SLD Patients Without Advanced Hepatic Fibrosis: Evidence From the NHANES 2017–2020 Database
Source: Can J Gastroenterol Hepatol. 2025 Aug 11;2025:6969761. doi: 10.1155/cjgh/6969761 (PMC12360881; doi:10.1155/cjgh/6969761)
Supplement: Supporting Information 12 — Supporting Figure 12: Association of CAP and LSM with spine BMD, BMC, and bone area stratified by glucose status. [file 6969761.f12.pptx]

## Slide 1
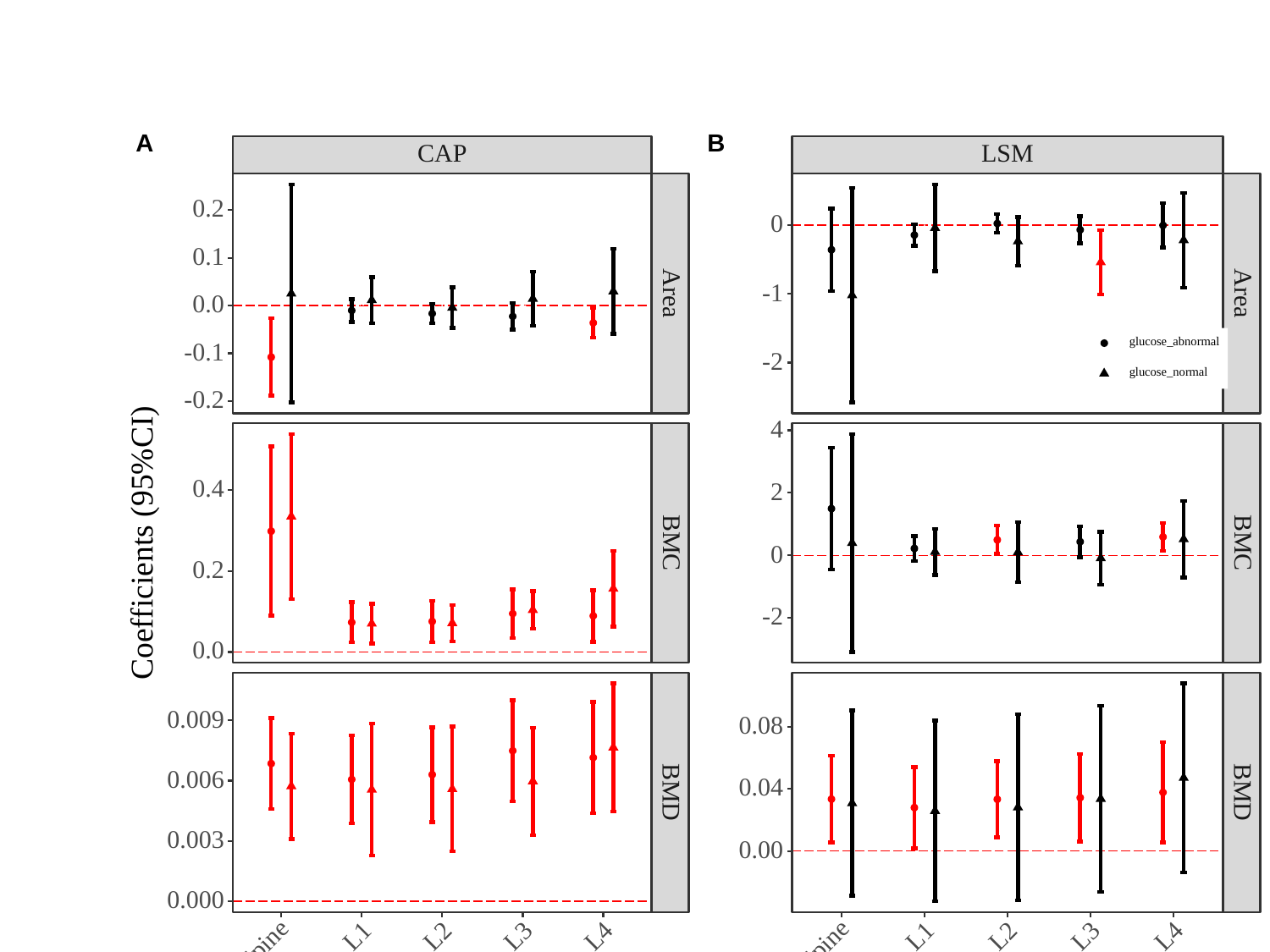

A
B
CAP
LSM
*
*
*
*
0.2
*
*
0
*
*
*
*
*
0.1
*
*
*
Area
Area
-1
*
0.0
*
*
*
*
*
glucose_abnormal
-0.1
-2
glucose_normal
-0.2
4
*
*
*
*
0.4
2
*
*
*
Coefficients (95%CI)
*
*
BMC
BMC
*
*
*
0
*
0.2
*
*
*
*
*
-2
*
*
0.0
*
*
*
*
*
0.009
*
0.08
*
*
*
*
*
*
*
*
*
*
*
*
*
0.006
*
0.04
BMD
BMD
0.003
0.00
0.000
L3
L3
L1
L2
L4
L1
L2
L4
Spine
Spine
