# Supplementary material for: Increased BMD in SLD Patients Without Advanced Hepatic Fibrosis: Evidence From the NHANES 2017–2020 Database
Source: Can J Gastroenterol Hepatol. 2025 Aug 11;2025:6969761. doi: 10.1155/cjgh/6969761 (PMC12360881; doi:10.1155/cjgh/6969761)
Supplement: Supporting Information 16 — Supporting Figure 16: Exposure–response relationship of CAP and LSM with spine BMD. [file 6969761.f16.pptx]

## Slide 1
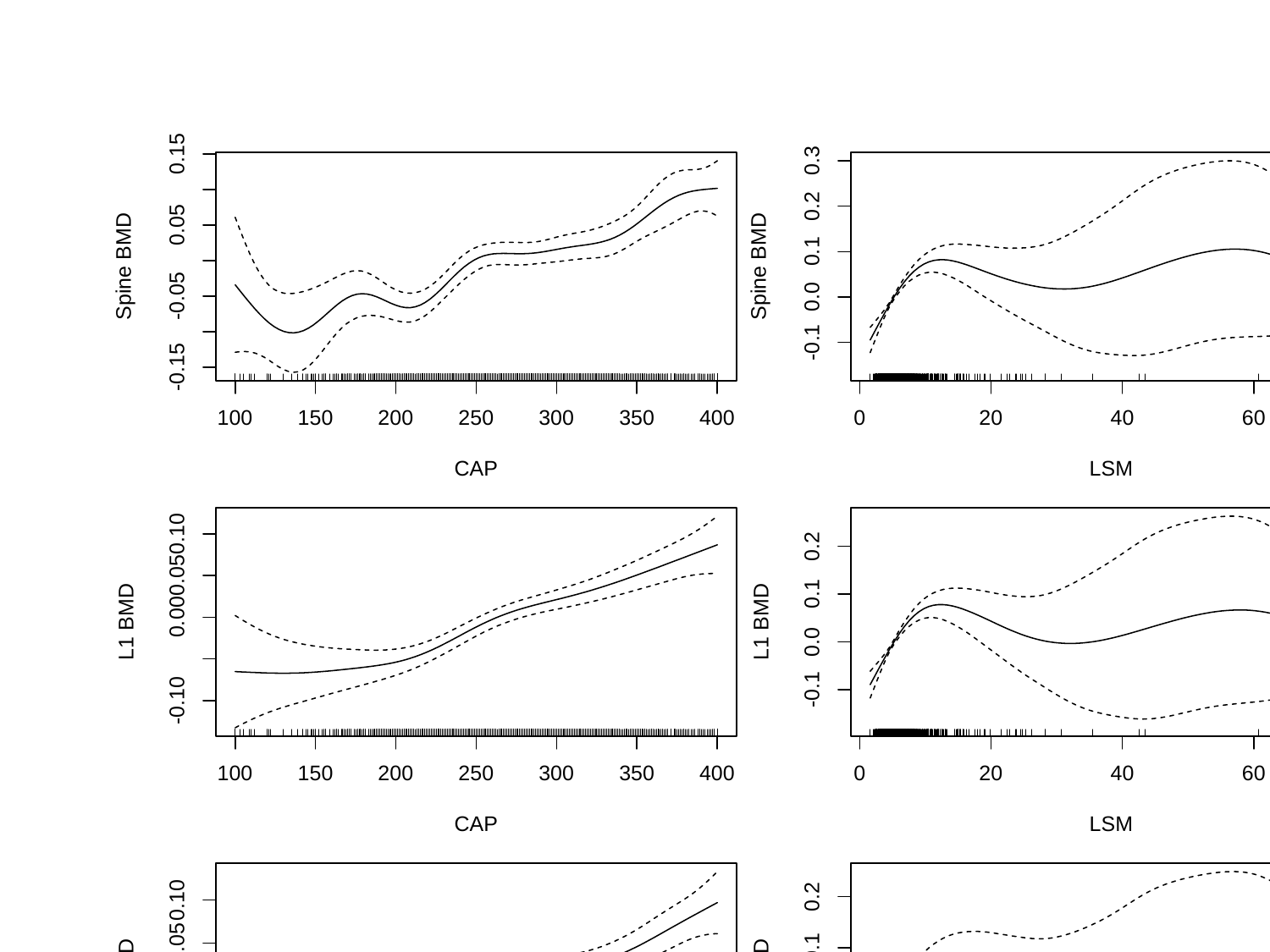

0.15
0.3
0.2
0.05
0.1
Spine BMD
Spine BMD
-0.05
0.0
-0.1
-0.15
60
200
250
20
400
40
300
350
100
150
0
LSM
CAP
0.10
0.2
0.05
0.1
0.00
L1 BMD
L1 BMD
0.0
-0.1
-0.10
60
200
250
20
400
40
300
350
100
150
0
LSM
CAP
0.2
0.10
0.05
0.1
L2 BMD
L2 BMD
0.00
0.0
-0.1
-0.10
60
200
250
20
400
40
300
350
100
150
0
LSM
CAP
0.3
0.15
0.2
0.05
0.1
L3 BMD
L3 BMD
0.0
-0.05
-0.1
-0.15
-0.2
60
200
250
20
400
40
300
350
100
150
0
LSM
CAP
0.4
0.1
0.3
0.2
0.0
L4 BMD
L4 BMD
0.1
0.0
-0.1
-0.2
-0.2
60
200
250
20
400
40
300
350
100
150
0
LSM
CAP
