# Supplementary material for: Increased BMD in SLD Patients Without Advanced Hepatic Fibrosis: Evidence From the NHANES 2017–2020 Database
Source: Can J Gastroenterol Hepatol. 2025 Aug 11;2025:6969761. doi: 10.1155/cjgh/6969761 (PMC12360881; doi:10.1155/cjgh/6969761)
Supplement: Supporting Information 17 — Supporting Table 1: Summary of participants with spine data stratified by SLD subcategories. Supporting Table 2: Summary of the spine healthy indicators stratified by SLD subcategories. Supporting Table 3: Association of SLD subcategories with spine BMD, BMC, and bone area. Supporting Table 4: Association of SLD subcategories with femur BMD, BMC, and bone area stratified by age. Supporting Table 5: Association of SLD subcategories with femur BMD, BMC, and bone area stratified by sex. Supporting Table 6: Association of SLD subcategories with femur BMD, BMC, and bone area stratified by BMI. Supporting Table 7: Association of SLD subcategories with femur BMD, BMC, and bone area stratified by glucose status. Supporting Table 8: Association of SLD subcategories with femur BMD, BMC, and bone area stratified by TG. Supporting Table 9: Association of SLD subcategories with femur BMD, BMC, and bone area stratified by HDL. Supporting Table 10: Association of SLD subcategories with femur BMD, BMC, and bone area stratified by LSM. Supporting Table 11: Association of SLD subcategories with spine BMD, BMC, and bone area stratified by age. Supporting Table 12: Association of SLD subcategories with spine BMD, BMC, and bone area stratified by sex. Supporting Table 13: Association of SLD subcategories with spine BMD, BMC, and bone area stratified by BMI. Supporting Table 14: Association of SLD subcategories with spine BMD, BMC, and bone area stratified by glucose status. Supporting Table 15: Association of SLD subcategories with spine BMD, BMC, and bone area stratified by TG. Supporting Table 16: Association of SLD subcategories with spine BMD, BMC, and bone area stratified by HDL. Supporting Table 17: Association of SLD subcategories with spine BMD, BMC, and bone area stratified by LSM. [file 6969761.f17.docx]

Supplementary table 1. Summary of participants with spine data stratified by SLD subcategories.

| Variable | Normal  (n=1011) | MASLD  (n=436) | MetALD  (n=106) | ALD  (n=49) | Total  (n=1602) | *p* |
| --- | --- | --- | --- | --- | --- | --- |
| Gender |  |  |  |  |  | <0.001 |
| Male | 419 (41.4%) | 221 (50.7%) | 49 (46.2%) | 33 (67.3%) | 722 (45.1%) |  |
| female | 592 (58.6%) | 215 (49.3%) | 57 (53.8%) | 16 (32.7%) | 880 (54.9%) |  |
| Age, years |  |  |  |  |  | <0.001 |
| Mean (SD) | 63.1 (8.80) | 62.7 (8.32) | 60.6 (6.72) | 58.4 (6.26) | 62.7 (8.53) |  |
| Median [Min, Max] | 62.0  [50.0, 80.0] | 62.0  [50.0, 80.0] | 60.0  [50.0, 80.0] | 58.0  [50.0, 80.0] | 61.0  [50.0, 80.0] |  |
| Race |  |  |  |  |  | <0.001 |
| Hispanic | 200 (19.8%) | 112 (25.7%) | 29 (27.4%) | 27 (55.1%) | 368 (23.0%) |  |
| Non-Hispanic White | 347 (34.3%) | 157 (36.0%) | 41 (38.7%) | 15 (30.6%) | 560 (35.0%) |  |
| Non-Hispanic Black | 277 (27.4%) | 98 (22.5%) | 27 (25.5%) | 5 (10.2%) | 407 (25.4%) |  |
| Other Race | 187 (18.5%) | 69 (15.8%) | 9 (8.5%) | 2 (4.1%) | 267 (16.7%) |  |
| Cotinine |  |  |  |  |  | 0.009 |
| Yes | 583 (57.7%) | 245 (56.2%) | 68 (64.2%) | 39 (79.6%) | 935 (58.4%) |  |
| No | 428 (42.3%) | 191 (43.8%) | 38 (35.8%) | 10 (20.4%) | 667 (41.6%) |  |
| Season |  |  |  |  |  | 0.250 |
| Cold | 488 (48.3%) | 222 (50.9%) | 56 (52.8%) | 30 (61.2%) | 796 (49.7%) |  |
| Warm | 523 (51.7%) | 214 (49.1%) | 50 (47.2%) | 19 (38.8%) | 806 (50.3%) |  |
| Education level |  |  |  |  |  | 0.008 |
| Less than 12th grade | 171 (16.9%) | 84 (19.3%) | 18 (17.0%) | 10 (20.4%) | 283 (17.7%) |  |
| High school graduate | 239 (23.6%) | 104 (23.9%) | 27 (25.5%) | 18 (36.7%) | 388 (24.2%) |  |
| Some college | 310 (30.7%) | 149 (34.2%) | 43 (40.6%) | 17 (34.7%) | 519 (32.4%) |  |
| College graduate or above | 291 (28.8%) | 99 (22.7%) | 18 (17.0%) | 4 (8.2%) | 412 (25.7%) |  |
| Marital status |  |  |  |  |  | 0.005 |
| Married | 581 (57.5%) | 293 (67.2%) | 59 (55.7%) | 30 (61.2%) | 963 (60.1%) |  |
| Unmarried | 430 (42.5%) | 143 (32.8%) | 47 (44.3%) | 19 (38.8%) | 639 (39.9%) |  |
| PIR |  |  |  |  |  | 0.596 |
| Mean (SD) | 2.86 (1.65) | 2.73 (1.62) | 2.88 (1.70) | 2.70 (1.74) | 2.82 (1.65) |  |
| Median [Min, Max] | 2.59  [0, 5.00] | 2.39  [0.0600, 5.00] | 2.60  [0, 5.00] | 2.34  [0, 5.00] | 2.48  [0, 5.00] |  |
| BMI, kg/m^2^ |  |  |  |  |  | <0.001 |
| Mean (SD) | 27.3 (5.24) | 32.7 (6.83) | 32.6 (6.36) | 33.7 (6.18) | 29.3 (6.38) |  |
| Median [Min, Max] | 26.5  [15.1, 48.0] | 31.5  [18.3, 62.1] | 31.2  [23.6, 55.0] | 33.9  [22.7, 50.7] | 28.3  [15.1, 62.1] |  |
| Waist, cm |  |  |  |  |  | <0.001 |
| Mean (SD) | 95.7 (12.6) | 109 (14.5) | 109 (13.4) | 114 (13.8) | 101 (14.9) |  |
| Median [Min, Max] | 94.8  [65.0, 145] | 108  [70.3, 154] | 106  [86.7, 154] | 112  [83.3, 147] | 99.6  [65.0, 154] |  |
| SBP, mmHg |  |  |  |  |  | 0.341 |
| Mean (SD) | 131 (19.3) | 131 (17.4) | 133 (19.1) | 135 (22.7) | 131 (18.9) |  |
| Median [Min, Max] | 129  [86.3, 200] | 129  [89.3, 195] | 132  [92.7, 188] | 134  [90.3, 187] | 129  [86.3, 200] |  |
| DBP, mmHg |  |  |  |  |  | <0.001 |
| Mean (SD) | 74.7 (10.8) | 75.9 (11.2) | 79.3 (12.2) | 80.1 (13.2) | 75.5 (11.2) |  |
| Median [Min, Max] | 74.3  [41.7, 121] | 75.7  [46.0, 117] | 79.2  [48.7, 122] | 78.0  [49.0, 107] | 75.0  [41.7, 122] |  |
| HDL-c, mmol/L |  |  |  |  |  | <0.001 |
| Mean (SD) | 1.51 (0.427) | 1.26 (0.345) | 1.36 (0.387) | 1.30 (0.380) | 1.43 (0.417) |  |
| Median [Min, Max] | 1.45  [0.130, 3.80] | 1.19  [0.590, 2.64] | 1.31  [0.700, 2.51] | 1.24  [0.570, 2.28] | 1.37  [0.130, 3.80] |  |
| TG, mmol/L |  |  |  |  |  | <0.001 |
| Mean (SD) | 1.46 (1.43) | 1.97 (1.29) | 2.20 (2.21) | 1.98 (1.01) | 1.66 (1.47) |  |
| Median [Min, Max] | 1.20  [0.418, 33.0] | 1.61  [0.418, 14.3] | 1.78  [0.508, 17.3] | 1.84  [0.531, 4.62] | 1.36  [0.418, 33.0] |  |
| Fasting glucose, mmol/L |  |  |  |  |  | <0.001 |
| Mean (SD) | 6.03 (1.91) | 7.52 (3.15) | 6.29 (1.56) | 6.80 (2.30) | 6.47 (2.39) |  |
| Median [Min, Max] | 5.66  [2.94, 25.0] | 6.44  [2.66, 22.2] | 6.28  [3.83, 15.3] | 6.14  [4.16, 13.3] | 5.83  [2.66, 25.0] |  |
| LSM, kPa |  |  |  |  |  | <0.001 |
| Mean (SD) | 5.55 (5.59) | 7.04 (5.56) | 6.07 (2.85) | 8.16 (6.27) | 6.07 (5.51) |  |
| Median [Min, Max] | 4.70  [2.10, 75.0] | 5.90  [1.60, 75.0] | 5.50  [2.50, 25.3] | 6.30  [2.50, 43.5] | 5.10  [1.60, 75.0] |  |
| CAP, dB/m |  |  |  |  |  | <0.001 |
| Mean (SD) | 234 (37.4) | 332 (34.1) | 327 (29.7) | 337 (33.1) | 270 (59.1) | <0.001 |
| Median [Min, Max] | 240  [100, 287] | 325  [288, 400] | 321  [288, 400] | 334  [288, 400] | 269  [100, 400] |  |

Abbreviations: MASLD: metabolic dysfunction-associated steatotic liver disease; MetALD: metabolic alcohol-related liver disease; ALD: alcoholic liver disease; PIR: poverty to income ratio; BMI: body mass index; SBP: systolic blood pressure; DBP: diastolic blood pressure; ‌HDL-c: high-density lipoprotein-cholesterol; TG: triglyceride; LSM: liver stiffness measurement; CAP: controlled attenuation parameter.

Supplementary table 2. Summary of the spine healthy indicators stratified by SLD subcategories.

| Variable | Normal | MASLD | MetALD | ALD | Total | *p* |
| --- | --- | --- | --- | --- | --- | --- |
|  | (n=1011) | (n=436) | (n=106) | (n=49) | (n=1602) |  |
| Spine BMD |  |  |  |  |  | <0.001 |
| Mean (SD) | 0.987 (0.170) | 1.04 (0.178) | 1.06 (0.183) | 1.09 (0.153) | 1.01 (0.175) |  |
| Median [Min, Max] | 0.985 [0.454, 1.64] | 1.04 [0.547, 1.72] | 1.02 [0.724, 1.80] | 1.08 [0.721, 1.58] | 1.00 [0.454, 1.80] |  |
| Spine BMC |  |  |  |  |  | <0.001 |
| Mean (SD) | 59.6 (17.1) | 62.8 (17.3) | 63.4 (15.3) | 69.7 (15.4) | 61.0 (17.1) |  |
| Median [Min, Max] | 57.7 [10.2, 140] | 60.9 [25.9, 141] | 61.0 [37.0, 110] | 70.8 [11.8, 111] | 59.7 [10.2, 141] |  |
| Spine area |  |  |  |  |  | 0.041 |
| Mean (SD) | 59.7 (9.85) | 60.0 (9.30) | 59.7 (7.42) | 63.6 (9.52) | 59.9 (9.57) |  |
| Median [Min, Max] | 59.0 [14.1, 96.5] | 59.8 [26.7, 86.9] | 60.0 [36.1, 79.7] | 64.5 [16.4, 77.4] | 59.6 [14.1, 96.5] |  |
| L1 BMD |  |  |  |  |  | <0.001 |
| Mean (SD) | 0.927 (0.170) | 0.983 (0.175) | 1.00 (0.174) | 1.02 (0.144) | 0.950 (0.174) |  |
| Median [Min, Max] | 0.919 [0, 1.68] | 0.975 [0.475, 1.63] | 0.985 [0.674, 1.45] | 1.00 [0.653, 1.48] | 0.941 [0, 1.68] |  |
| L1 BMC |  |  |  |  |  | <0.001 |
| Mean (SD) | 12.7 (3.78) | 13.6 (3.81) | 13.6 (3.52) | 14.7 (3.25) | 13.1 (3.78) |  |
| Median [Min, Max] | 12.2 [0, 29.4] | 13.2 [5.22, 30.4] | 13.2 [7.87, 26.9] | 14.7 [6.58, 25.7] | 12.6 [0, 30.4] |  |
| L1 area |  |  |  |  |  | 0.061 |
| Mean (SD) | 13.5 (2.18) | 13.6 (2.04) | 13.5 (1.69) | 14.4 (1.88) | 13.6 (2.11) |  |
| Median [Min, Max] | 13.3 [0, 23.5] | 13.6 [8.50, 19.5] | 13.5 [10.4, 19.1] | 14.5 [9.20, 19.7] | 13.4 [0, 23.5] |  |
| L2 BMD |  |  |  |  |  | <0.001 |
| Mean (SD) | 0.984 (0.176) | 1.03 (0.182) | 1.05 (0.188) | 1.09 (0.152) | 1.00 (0.180) |  |
| Median [Min, Max] | 0.974 [0.461, 1.62] | 1.03 [0.542, 1.71] | 1.02 [0.703, 1.80] | 1.10 [0.708, 1.56] | 1.00 [0.461, 1.80] |  |
| L2 BMC |  |  |  |  |  | <0.001 |
| Mean (SD) | 14.2 (4.05) | 15.1 (4.31) | 15.3 (4.48) | 16.6 (3.45) | 14.6 (4.16) |  |
| Median [Min, Max] | 13.7 [5.37, 33.4] | 14.5 [6.64, 32.7] | 15.0 [8.05, 38.5] | 16.7 [5.26, 24.4] | 14.2 [5.26, 38.5] |  |
| L2 area |  |  |  |  |  | 0.056 |
| Mean (SD) | 14.3 (2.21) | 14.4 (2.23) | 14.4 (1.96) | 15.2 (2.07) | 14.4 (2.20) |  |
| Median [Min, Max] | 14.2 [7.88, 23.3] | 14.3 [7.52, 22.2] | 14.2 [10.6, 21.5] | 15.4 [7.23, 18.5] | 14.3 [7.23, 23.3] |  |
| L3 BMD |  |  |  |  |  | <0.001 |
| Mean (SD) | 1.02 (0.176) | 1.07 (0.191) | 1.09 (0.203) | 1.12 (0.153) | 1.04 (0.184) |  |
| Median [Min, Max] | 1.01 [0.464, 1.71] | 1.06 [0.574, 1.88] | 1.06 [0.706, 2.04] | 1.12 [0.718, 1.58] | 1.03 [0.464, 2.04] |  |
| L3 BMC |  |  |  |  |  | <0.001 |
| Mean (SD) | 16.1 (4.46) | 16.9 (4.68) | 17.2 (5.03) | 18.6 (3.51) | 16.5 (4.56) |  |
| Median [Min, Max] | 15.6 [4.76, 40.0] | 16.2 [6.72, 38.8] | 16.2 [9.02, 44.0] | 18.9 [10.8, 27.3] | 16.0 [4.76, 44.0] |  |
| L3 area |  |  |  |  |  | 0.048 |
| Mean (SD) | 15.6 (2.36) | 15.7 (2.35) | 15.7 (2.13) | 16.6 (1.86) | 15.7 (2.33) |  |
| Median [Min, Max] | 15.4 [9.67, 24.3] | 15.6 [9.85, 25.3] | 15.6 [9.76, 21.6] | 16.7 [11.6, 19.7] | 15.5 [9.67, 25.3] |  |
| L4 BMD |  |  |  |  |  | <0.001 |
| Mean (SD) | 1.02 (0.184) | 1.07 (0.213) | 1.08 (0.187) | 1.11 (0.161) | 1.04 (0.194) |  |
| Median [Min, Max] | 1.01 [0.452, 1.94] | 1.05 [0.560, 2.85] | 1.04 [0.763, 1.80] | 1.10 [0.792, 1.66] | 1.03 [0.452, 2.85] |  |
| L4 BMC |  |  |  |  |  | <0.001 |
| Mean (SD) | 17.4 (5.11) | 18.5 (5.56) | 18.3 (4.96) | 20.2 (4.50) | 17.8 (5.25) |  |
| Median [Min, Max] | 16.9 [5.43, 43.4] | 18.0 [7.23, 59.8] | 17.4 [10.5, 39.1] | 20.2 [12.4, 33.3] | 17.3 [5.43, 59.8] |  |
| L4 area |  |  |  |  |  | 0.232 |
| Mean (SD) | 16.9 (2.79) | 17.1 (2.61) | 16.8 (2.48) | 18.1 (2.34) | 17.0 (2.71) |  |
| Median [Min, Max] | 16.6 [9.35, 27.5] | 16.8 [10.9, 25.2] | 16.7 [11.7, 24.2] | 18.3 [13.6, 23.4] | 16.7 [9.35, 27.5] |  |

Abbreviations: MASLD: metabolic dysfunction-associated steatotic liver disease; MetALD: metabolic alcohol-related liver disease; ALD: alcoholic liver disease; BMD: bone mineral density; BMC: bone mineral content.

Supplementary table 3. Association of SLD subcategories with spine BMD, BMC and bone area.

|  |  | Normal | MASLD | MetALD | ALD |
| --- | --- | --- | --- | --- | --- |
|  |  |  | β (95% CI) | β (95% CI) | β (95% CI) |
| Total spine | BMD | Reference | **0.061 (0.027, 0.095)** | **0.068 (0.031, 0.105)** | **0.070 (0.024, 0.116)** |
|  | BMC | Reference | **3.272 (0.894, 5.650)** | 2.992 (-0.690, 6.673) | **5.984 (1.748, 10.220)** |
|  | Area | Reference | -0.303 (-1.983, 1.377) | -0.659 (-2.740, 1.422) | 1.601 (-0.571, 3.772) |
| L1 | BMD | Reference | **0.064 (0.034, 0.094)** | **0.061 (0.031, 0.090)** | **0.049 (0.002, 0.096)** |
|  | BMC | Reference | **0.918 (0.375, 1.461)** | 0.664 (-0.061, 1.389) | 0.485 (-0.685, 1.655) |
|  | Area | Reference | 0.084 (-0.146, 0.314) | -0.073 (-0.593, 0.448) | -0.072 (-0.747, 0.603) |
| L2 | BMD | Reference | **0.056 (0.019, 0.093)** | **0.070 (0.031, 0.108)** | **0.082 (0.038, 0.126)** |
|  | BMC | Reference | **0.734 (0.059, 1.408)** | 0.954 (-0.022, 1.929) | **1.502 (0.499, 2.504)** |
|  | Area | Reference | -0.089 (-0.372, 0.193) | -0.026 (-0.541, 0.489) | 0.286 (-0.204, 0.775) |
| L3 | BMD | Reference | **0.066 (0.026, 0.106)** | **0.077 (0.035, 0.120)** | **0.087 (0.040, 0.135)** |
|  | BMC | Reference | **0.938 (0.127, 1.748)** | 1.156 (-0.042, 2.354) | **1.631 (0.549, 2.714)** |
|  | Area | Reference | -0.107 (-0.522, 0.308) | -0.042 (-0.604, 0.521) | 0.208 (-0.252, 0.668) |
| L4 | BMD | Reference | **0.064 (0.030, 0.098)** | **0.067 (0.020, 0.113)** | **0.059 (0.002, 0.115)** |
|  | BMC | Reference | **1.230 (0.508, 1.953)** | 0.536 (-0.739, 1.811) | **1.868 (0.526, 3.209)** |
|  | Area | Reference | 0.124 (-0.291, 0.539) | -0.467 (-1.118, 0.184) | 0.861 (-0.009, 1.731) |

Note: Boldface type indicates statistical significance.

Abbreviations: MASLD: metabolic dysfunction-associated steatotic liver disease; MetALD: metabolic alcohol-related liver disease; ALD: alcoholic liver disease; BMD: bone mineral density; BMC: bone mineral content.

Supplementary table 4. Association of SLD subcategories with femur BMD, BMC and bone area stratified by age.

|  | | Normal | MASLD  β (95% CI) | MetALD  β (95% CI) | ALD  β (95% CI) |
| --- | --- | --- | --- | --- | --- |
| **Age<65** |  |  |  |  |  |
| Total femur | BMD | Reference | **0.048 (0.029, 0.068)** | 0.054 (-0.004, 0.112) | **0.041 (0.003, 0.080)** |
|  | BMC | Reference | **2.052 (0.910, 3.194)** | 1.569 (-1.104, 4.241) | 1.378 (-0.783, 3.538) |
|  | Area | Reference | 0.204 (-0.544, 0.952) | -0.264 (-1.110, 0.582) | -0.039 (-1.541, 1.464) |
| Femur neck | BMD | Reference | **0.031 (0.009, 0.053)** | 0.041 (-0.012, 0.093) | 0.034 (-0.010, 0.078) |
|  | BMC | Reference | **0.192 (0.059, 0.326)** | 0.223 (-0.106, 0.551) | 0.153 (-0.081, 0.386) |
|  | Area | Reference | 0.037 (-0.039, 0.113) | 0.033 (-0.078, 0.144) | -0.014 (-0.150, 0.122) |
| Trochanter | BMD | Reference | **0.036 (0.011, 0.061)** | **0.048 (0.000, 0.095)** | **0.045 (0.013, 0.077)** |
|  | BMC | Reference | **0.420 (0.148, 0.691)** | 0.448 (-0.224, 1.121) | 0.163 (-0.552, 0.878) |
|  | Area | Reference | -0.008 (-0.310, 0.295) | -0.102 (-0.402, 0.198) | -0.448 (-1.141, 0.245) |
| Intertrochanter | BMD | Reference | **0.059 (0.041, 0.078)** | 0.061 (-0.005, 0.127) | 0.034 (-0.014, 0.081) |
|  | BMC | Reference | **1.439 (0.586, 2.293)** | 0.899 (-0.909, 2.706) | 1.061 (-0.272, 2.395) |
|  | Area | Reference | 0.175 (-0.345, 0.695) | -0.195 (-0.883, 0.493) | 0.424 (-0.390, 1.239) |
| Wards triangle | BMD | Reference | **0.036 (0.012, 0.060)** | 0.013 (-0.054, 0.080) | 0.009 (-0.051, 0.069) |
|  | BMC | Reference | **0.046 (0.012, 0.081)** | 0.010 (-0.078, 0.097) | -0.004 (-0.085, 0.078) |
|  | Area | Reference | 0.007 (-0.013, 0.026) | -0.014 (-0.040, 0.012) | -0.020 (-0.044, 0.003) |
| **Age≥65** |  |  |  |  |  |
| Total femur | BMD | Reference | **0.066 (0.036, 0.097)** | **0.078 (0.036, 0.120)** | 0.022 (-0.060, 0.105) |
|  | BMC | Reference | **2.530 (0.976, 4.083)** | **2.827 (0.505, 5.149)** | 2.453 (-1.448, 6.355) |
|  | Area | Reference | -0.037 (-0.817, 0.742) | -0.104 (-1.671, 1.462) | 1.809 (-3.046, 6.664) |
| Femur neck | BMD | Reference | **0.040 (0.012, 0.069)** | **0.059 (0.013, 0.105)** | -0.002 (-0.092, 0.088) |
|  | BMC | Reference | **0.225 (0.060, 0.390)** | **0.299 (0.073, 0.524)** | 0.033 (-0.353, 0.419) |
|  | Area | Reference | 0.019 (-0.051, 0.090) | 0.006 (-0.073, 0.086) | 0.089 (-0.308, 0.487) |
| Trochanter | BMD | Reference | **0.050 (0.026, 0.075)** | **0.057 (0.020, 0.094)** | 0.058 (-0.027, 0.144) |
|  | BMC | Reference | **0.446 (0.011, 0.881)** | **0.688 (0.043, 1.332)** | 0.730 (-0.290, 1.751) |
|  | Area | Reference | -0.227 (-0.608, 0.154) | 0.041 (-0.594, 0.677) | 0.057 (-1.767, 1.882) |
| Intertrochanter | BMD | Reference | **0.080 (0.045, 0.115)** | **0.097 (0.050, 0.143)** | -0.004 (-0.100, 0.092) |
|  | BMC | Reference | **1.859 (0.788, 2.929)** | **1.841 (0.235, 3.447)** | 1.691 (-1.151, 4.533) |
|  | Area | Reference | 0.172 (-0.373, 0.717) | -0.151 (-1.195, 0.893) | 1.670 (-1.102, 4.441) |
| Wards triangle | BMD | Reference | **0.035 (0.003, 0.068)** | **0.047 (0.000, 0.094)** | -0.005 (-0.051, 0.041) |
|  | BMC | Reference | 0.040 (-0.000, 0.081) | 0.060 (-0.005, 0.124) | 0.020 (-0.038, 0.077) |
|  | Area | Reference | -0.001 (-0.014, 0.012) | 0.008 (-0.017, 0.033) | 0.049 (-0.024, 0.122) |

Note: Boldface type indicates statistical significance.

Abbreviations: MASLD: metabolic dysfunction-associated steatotic liver disease; MetALD: metabolic alcohol-related liver disease; ALD: alcoholic liver disease; BMD: bone mineral density; BMC: bone mineral content.

Supplementary table 5. Association of SLD subcategories with femur BMD, BMC and bone area stratified by gender.

|  |  | Normal | MASLD | MetALD | ALD |
| --- | --- | --- | --- | --- | --- |
|  |  |  | β (95% CI) | β (95% CI) | β (95% CI) |
| **Male** |  |  |  |  |  |
| Total femur | BMD | Reference | **0.049 (0.024, 0.074)** | 0.037 (-0.020, 0.093) | 0.023 (-0.015, 0.061) |
|  | BMC | Reference | **1.914 (0.501, 3.326)** | 0.685 (-2.343, 3.714) | 0.763 (-1.620, 3.145) |
|  | Area | Reference | -0.274 (-1.053, 0.504) | -0.962 (-2.227, 0.302) | -0.139 (-1.599, 1.322) |
| Femur neck | BMD | Reference | **0.030 (0.001, 0.058)** | 0.016 (-0.038, 0.071) | 0.011 (-0.040, 0.063)* |
|  | BMC | Reference | **0.189 (0.023, 0.355)** | 0.118 (-0.209, 0.446) | 0.012 (-0.262, 0.286)* |
|  | Area | Reference | 0.021 (-0.069, 0.111) | 0.036 (-0.072, 0.143) | -0.044 (-0.229, 0.141) |
| Trochanter | BMD | Reference | **0.037 (0.017, 0.058)** | 0.026 (-0.020, 0.072) | **0.040 (0.004, 0.075)** |
|  | BMC | Reference | 0.298 (-0.114, 0.710) | 0.238 (-0.551, 1.026) | 0.058 (-0.637, 0.754) |
|  | Area | Reference | -0.279 (-0.626, 0.067) | -0.155 (-0.641, 0.331) | -0.564 (-1.103, -0.026) |
| Intertrochanter | BMD | Reference | **0.058 (0.027, 0.089)** | 0.053 (-0.016, 0.123) | 0.008 (-0.036, 0.053)* |
|  | BMC | Reference | **1.426 (0.481, 2.370)** | 0.330 (-1.814, 2.474) | 0.692 (-0.870, 2.253) |
|  | Area | Reference | -0.016 (-0.528, 0.496) | -0.842 (-1.774, 0.090) | 0.471 (-0.447, 1.389) |
| Wards triangle | BMD | Reference | **0.033 (0.008, 0.059)** | -0.023 (-0.098, 0.051) | -0.014 (-0.066, 0.038)* |
|  | BMC | Reference | **0.036 (0.006, 0.066)** | -0.034 (-0.127, 0.059) | -0.029 (-0.091, 0.033)* |
|  | Area | Reference | -0.002 (-0.012, 0.008) | -0.012 (-0.038, 0.014) | -0.011 (-0.034, 0.011) |
| **Female** |  |  |  |  |  |
| Total femur | BMD | Reference | **0.064 (0.029, 0.098)** | **0.073 (0.020, 0.126)** | **0.096 (0.028, 0.164)** |
|  | BMC | Reference | **2.463 (1.133, 3.794)** | **2.654 (0.731, 4.577)** | **4.294 (1.576, 7.011)** |
|  | Area | Reference | 0.337 (-0.139, 0.813) | 0.309 (-0.426, 1.043) | 1.285 (-0.233, 2.804) |
| Femur neck | BMD | Reference | **0.040 (0.008, 0.073)** | **0.063 (0.013, 0.113)** | **0.084 (0.035, 0.133)** |
|  | BMC | Reference | **0.221 (0.061, 0.382)** | **0.329 (0.045, 0.613)** | **0.518 (0.214, 0.823)** |
|  | Area | Reference | 0.034 (-0.034, 0.102) | 0.032 (-0.078, 0.141) | 0.143 (-0.041, 0.327) |
| Trochanter | BMD | Reference | **0.047 (0.015, 0.078)** | **0.065 (0.022, 0.109)** | 0.065 (-0.013, 0.143) |
|  | BMC | Reference | **0.534 (0.225, 0.843)** | **0.715 (0.248, 1.182)** | **0.951 (0.025, 1.878)** |
|  | Area | Reference | 0.038 (-0.173, 0.249) | 0.016 (-0.249, 0.281) | 0.416 (-0.463, 1.295) |
| Intertrochanter | BMD | Reference | **0.079 (0.043, 0.115)** | **0.077 (0.017, 0.137)** | **0.118 (0.046, 0.191)** |
|  | BMC | Reference | **1.709 (0.793, 2.624)** | **1.611 (0.332, 2.890)** | **2.826 (1.215, 4.438)** |
|  | Area | Reference | 0.266 (-0.140, 0.672) | 0.261 (-0.364, 0.885) | 0.728 (-0.185, 1.641) |
| Wards triangle | BMD | Reference | 0.036 (-0.002, 0.073) | 0.044 (-0.019, 0.106) | **0.092 (0.017, 0.168)** |
|  | BMC | Reference | 0.047 (-0.002, 0.095) | 0.050 (-0.038, 0.139) | **0.122 (0.006, 0.239)** |
|  | Area | Reference | 0.007 (-0.011, 0.025) | -0.007 (-0.036, 0.022) | 0.011 (-0.053, 0.075) |

Note: Boldface type indicates statistical significance. An asterisk indicates that the product term of the interaction is significant.

Abbreviations: MASLD: metabolic dysfunction-associated steatotic liver disease; MetALD: metabolic alcohol-related liver disease; ALD: alcoholic liver disease; BMD: bone mineral density; BMC: bone mineral content.

Supplementary table 6. Association of SLD subcategories with femur BMD, BMC and bone area stratified by BMI.

|  |  | Normal | MASLD | MetALD | ALD |
| --- | --- | --- | --- | --- | --- |
|  |  |  | β (95% CI) | β (95% CI) | β (95% CI) |
| **BMI<25** |  |  |  |  |  |
| Total femur | BMD | Reference | **0.038 (0.002, 0.075)** | -0.007 (-0.053, 0.039) | 0.108 (-0.020, 0.235) |
|  | BMC | Reference | **1.349 (0.014, 2.685)** | -3.241 (-6.373, -0.108)* | 7.092 (3.206, 10.978)* |
|  | Area | Reference | -0.399 (-1.212, 0.415) | -3.611 (-6.896, -0.327) | 2.093 (-0.384, 4.571) |
| Femur neck | BMD | Reference | 0.029 (-0.006, 0.063) | 0.026 (-0.042, 0.094) | **0.089 (0.040, 0.138)*** |
|  | BMC | Reference | **0.247 (0.041, 0.452)** | -0.050 (-0.299, 0.199) | **0.661 (0.148, 1.174)*** |
|  | Area | Reference | 0.118 (0.017, 0.220)* | -0.267 (-0.522, -0.013)* | 0.152 (-0.261, 0.565) |
| Trochanter | BMD | Reference | **0.036 (0.008, 0.064)** | 0.001 (-0.047, 0.049) | **0.147 (0.053, 0.240)*** |
|  | BMC | Reference | 0.288 (-0.135, 0.711) | -0.243 (-1.499, 1.013) | 1.078 (-0.641, 2.797) |
|  | Area | Reference | -0.257 (-0.854, 0.340) | -0.285 (-2.461, 1.890) | **-0.977 (-1.842, -0.112)** |
| Intertrochanter | BMD | Reference | **0.044 (0.000, 0.088)** | -0.001 (-0.058, 0.055) | 0.074 (-0.116, 0.264) |
|  | BMC | Reference | 0.816 (-0.197, 1.829) | **-2.947 (-4.979, -0.915)*** | **5.353 (3.445, 7.261)*** |
|  | Area | Reference | -0.260 (-0.778, 0.257) | **-3.060 (-4.198, -1.923)*** | 2.909 (-0.414, 6.232) |
| Wards triangle | BMD | Reference | 0.029 (-0.018, 0.075) | **-0.060 (-0.101, -0.018)** | -0.025 (-0.214, 0.164) |
|  | BMC | Reference | 0.025 (-0.032, 0.082) | **-0.103 (-0.159, -0.047)*** | -0.034 (-0.260, 0.192) |
|  | Area | Reference | -0.003 (-0.023, 0.016) | **-0.068 (-0.101, -0.035)*** | 0.002 (-0.035, 0.039) |
| **BMI≥25** |  |  |  |  |  |
| Total femur | BMD | Reference | **0.032 (0.015, 0.048)** | 0.034 (-0.006, 0.074) | 0.008 (-0.031, 0.046) |
|  | BMC | Reference | **1.063 (0.081, 2.044)** | 0.900 (-0.789, 2.589) | 0.017 (-2.093, 2.128) |
|  | Area | Reference | -0.160 (-0.742, 0.422) | -0.264 (-1.087, 0.558) | -0.065 (-1.478, 1.348) |
| Femur neck | BMD | Reference | 0.013 (-0.007, 0.032) | 0.018 (-0.023, 0.060) | 0.000 (-0.044, 0.044) |
|  | BMC | Reference | 0.064 (-0.056, 0.184) | 0.107 (-0.124, 0.338) | -0.040 (-0.275, 0.196) |
|  | Area | Reference | -0.003 (-0.057, 0.050) | 0.030 (-0.043, 0.103) | -0.023 (-0.181, 0.134) |
| Trochanter | BMD | Reference | **0.021 (0.004, 0.038)** | 0.029 (-0.001, 0.060) | 0.021 (-0.012, 0.054) |
|  | BMC | Reference | 0.127 (-0.167, 0.420) | 0.282 (-0.149, 0.712) | -0.110 (-0.762, 0.542) |
|  | Area | Reference | -0.184 (-0.470, 0.103) | -0.074 (-0.387, 0.240) | -0.425 (-1.012, 0.161) |
| Intertrochanter | BMD | Reference | **0.040 (0.023, 0.058)** | 0.041 (-0.007, 0.089) | -0.005 (-0.054, 0.044) |
|  | BMC | Reference | **0.872 (0.183, 1.560)** | 0.512 (-0.715, 1.739) | 0.167 (-1.172, 1.506) |
|  | Area | Reference | 0.028 (-0.400, 0.456) | -0.220 (-0.876, 0.436) | 0.385 (-0.427, 1.197) |
| Wards triangle | BMD | Reference | 0.018 (-0.006, 0.041) | 0.000 (-0.057, 0.058) | -0.012 (-0.062, 0.037) |
|  | BMC | Reference | 0.024 (-0.004, 0.052) | -0.000 (-0.074, 0.074) | -0.021 (-0.083, 0.042) |
|  | Area | Reference | 0.004 (-0.007, 0.015) | -0.004 (-0.025, 0.017) | -0.006 (-0.031, 0.019) |

Note: Boldface type indicates statistical significance. An asterisk indicates that the product term of the interaction is significant.

Abbreviations: MASLD: metabolic dysfunction-associated steatotic liver disease; MetALD: metabolic alcohol-related liver disease; ALD: alcoholic liver disease; BMI: body mass index; BMD: bone mineral density; BMC: bone mineral content.

Supplementary table 7. Association of SLD subcategories with femur BMD, BMC and bone area stratified by glucose status.

|  |  | Normal | MASLD | MetALD | ALD |
| --- | --- | --- | --- | --- | --- |
|  |  |  | β (95% CI) | β (95% CI) | β (95% CI) |
| **Normal glucose** |  |  |  |  |  |
| Total femur | BMD | Reference | 0.027 (-0.001, 0.054) | **0.052 (0.012, 0.092)** | 0.044 (-0.012, 0.101) |
|  | BMC | Reference | 0.303 (-1.019, 1.625)* | **1.795 (0.085, 3.505)** | 2.269 (-1.786, 6.323) |
|  | Area | Reference | **-0.824 (-1.360, -0.287)*** | -0.091 (-1.074, 0.892) | 0.668 (-1.945, 3.281) |
| Femur neck | BMD | Reference | 0.018 (-0.009, 0.045) | 0.036 (-0.003, 0.075) | 0.034 (-0.025, 0.094) |
|  | BMC | Reference | 0.130 (-0.034, 0.295) | **0.292 (0.065, 0.520)** | 0.271 (-0.102, 0.645) |
|  | Area | Reference | 0.058 (-0.030, 0.146) | **0.145 (0.032, 0.258)*** | **0.114 (0.028, 0.200)** |
| Trochanter | BMD | Reference | **0.026 (0.003, 0.048)** | **0.052 (0.011, 0.092)** | 0.049 (-0.002, 0.101) |
|  | BMC | Reference | 0.116 (-0.264, 0.496) | 0.592 (-0.047, 1.230) | 0.189 (-1.285, 1.663) |
|  | Area | Reference | -0.268 (-0.560, 0.025) | -0.009 (-0.470, 0.451) | -0.515 (-1.918, 0.888) |
| Intertrochanter | BMD | Reference | 0.034 (-0.004, 0.071) | **0.057 (0.011, 0.103)** | 0.032 (-0.033, 0.098) |
|  | BMC | Reference | 0.057 (-0.828, 0.942)* | 0.913 (-0.223, 2.050) | 1.809 (-0.615, 4.232) |
|  | Area | Reference | **-0.612 (-1.067, -0.158)*** | -0.227 (-1.071, 0.617) | 1.068 (-0.283, 2.419) |
| Wards triangle | BMD | Reference | -0.001 (-0.044, 0.043) | -0.000 (-0.064, 0.063) | -0.001 (-0.094, 0.093) |
|  | BMC | Reference | -0.010 (-0.067, 0.047) | -0.013 (-0.104, 0.079) | -0.019 (-0.137, 0.098) |
|  | Area | Reference | -0.015 (-0.043, 0.012) | -0.027 (-0.065, 0.012) | **-0.034 (-0.061, -0.007)** |
| **Abnormal glucose** |  |  |  |  |  |
| Total femur | BMD | Reference | **0.054 (0.026, 0.082)** | **0.065 (0.008, 0.122)** | 0.029 (-0.017, 0.074) |
|  | BMC | Reference | **2.362 (1.026, 3.698)** | 2.035 (-0.357, 4.428) | 1.102 (-1.635, 3.839) |
|  | Area | Reference | 0.319 (-0.284, 0.922) | -0.203 (-0.963, 0.558) | 0.210 (-1.898, 2.319) |
| Femur neck | BMD | Reference | **0.032 (0.007, 0.058)** | 0.050 (-0.002, 0.101) | 0.018 (-0.031, 0.066) |
|  | BMC | Reference | **0.185 (0.026, 0.344)** | 0.210 (-0.105, 0.525) | 0.031 (-0.241, 0.303) |
|  | Area | Reference | 0.015 (-0.048, 0.078) | -0.040 (-0.126, 0.046) | -0.046 (-0.257, 0.165) |
| Trochanter | BMD | Reference | **0.038 (0.010, 0.065)** | **0.049 (0.005, 0.093)** | **0.039 (0.004, 0.074)** |
|  | BMC | Reference | **0.414 (0.033, 0.796)** | 0.500 (-0.032, 1.031) | 0.218 (-0.416, 0.852) |
|  | Area | Reference | -0.060 (-0.345, 0.226) | -0.061 (-0.309, 0.187) | -0.289 (-0.996, 0.418) |
| Intertrochanter | BMD | Reference | **0.065 (0.035, 0.095)** | **0.078 (0.011, 0.145)** | 0.019 (-0.040, 0.078) |
|  | BMC | Reference | **1.761 (0.892, 2.631)** | 1.325 (-0.288, 2.938) | 0.852 (-1.099, 2.803) |
|  | Area | Reference | 0.364 (-0.054, 0.782) | -0.101 (-0.661, 0.459) | 0.548 (-0.722, 1.818) |
| Wards triangle | BMD | Reference | **0.040 (0.013, 0.068)** | 0.028 (-0.036, 0.092) | 0.005 (-0.050, 0.061) |
|  | BMC | Reference | **0.051 (0.017, 0.086)** | 0.035 (-0.050, 0.119) | 0.004 (-0.071, 0.078) |
|  | Area | Reference | 0.009 (-0.003, 0.022) | 0.001 (-0.021, 0.024) | 0.004 (-0.031, 0.038) |

Note: Boldface type indicates statistical significance. An asterisk indicates that the product term of the interaction is significant.

Abbreviations: MASLD: metabolic dysfunction-associated steatotic liver disease; MetALD: metabolic alcohol-related liver disease; ALD: alcoholic liver disease; BMD: bone mineral density; BMC: bone mineral content.

Supplementary table 8. Association of SLD subcategories with femur BMD, BMC and bone area stratified by TG status.

|  |  | Normal | MASLD | MetALD | ALD |
| --- | --- | --- | --- | --- | --- |
|  |  |  | β (95% CI) | β (95% CI) | β (95% CI) |
| **Normal TG** |  |  |  |  |  |
| Total femur | BMD | Reference | **0.049 (0.029, 0.068)** | **0.058 (0.004, 0.112)** | **0.059 (0.014, 0.105)** |
|  | BMC | Reference | **1.858 (0.730, 2.986)** | 1.677 (-0.577, 3.931) | **3.617 (1.243, 5.991)*** |
|  | Area | Reference | 0.037 (-0.766, 0.840) | -0.400 (-1.263, 0.464) | 1.381 (0.189, 2.572) |
| Femur neck | BMD | Reference | **0.034 (0.015, 0.054)** | 0.040 (-0.009, 0.089) | 0.036 (-0.009, 0.082) |
|  | BMC | Reference | **0.195 (0.074, 0.317)** | 0.212 (-0.070, 0.495) | 0.289 (-0.020, 0.599) |
|  | Area | Reference | 0.029 (-0.036, 0.094) | 0.021 (-0.078, 0.120) | 0.125 (-0.019, 0.268)* |
| Trochanter | BMD | Reference | **0.038 (0.019, 0.057)** | **0.049 (0.007, 0.091)** | **0.064 (0.024, 0.104)** |
|  | BMC | Reference | **0.470 (0.157, 0.782)** | 0.508 (-0.089, 1.105) | **0.758 (0.011, 1.506)*** |
|  | Area | Reference | 0.046 (-0.304, 0.396) | -0.043 (-0.425, 0.340) | 0.019 (-0.644, 0.682) |
| Intertrochanter | BMD | Reference | **0.062 (0.039, 0.084)** | **0.070 (0.007, 0.134)** | 0.056 (-0.005, 0.116) |
|  | BMC | Reference | **1.193 (0.326, 2.059)** | 0.958 (-0.521, 2.437) | **2.571 (1.090, 4.052)*** |
|  | Area | Reference | -0.037 (-0.645, 0.571) | -0.379 (-0.982, 0.224) | **1.237 (0.501, 1.972)** |
| Wards triangle | BMD | Reference | 0.024 (-0.000, 0.048) | 0.032 (-0.038, 0.103) | 0.026 (-0.040, 0.093) |
|  | BMC | Reference | **0.034 (0.000, 0.069)** | 0.031 (-0.061, 0.123) | 0.028 (-0.068, 0.123) |
|  | Area | Reference | 0.010 (-0.010, 0.030) | -0.013 (-0.042, 0.015) | 0.001 (-0.037, 0.039) |
| **Abnormal TG** |  |  |  |  |  |
| Total femur | BMD | Reference | **0.044 (0.016, 0.072)** | 0.046 (-0.008, 0.101) | 0.013 (-0.043, 0.068) |
|  | BMC | Reference | **1.834 (0.472, 3.196)** | 1.415 (-1.639, 4.469) | -0.205 (-2.341, 1.930) |
|  | Area | Reference | 0.058 (-0.560, 0.676) | -0.221 (-1.739, 1.297) | -0.459 (-2.193, 1.275) |
| Femur neck | BMD | Reference | 0.023 (-0.010, 0.057) | 0.039 (-0.008, 0.085) | 0.020 (-0.041, 0.082) |
|  | BMC | Reference | 0.114 (-0.081, 0.309) | 0.186 (-0.111, 0.484) | -0.043 (-0.333, 0.247) |
|  | Area | Reference | -0.016 (-0.086, 0.053) | -0.001 (-0.124, 0.123) | -0.148 (-0.328, 0.032) |
| Trochanter | BMD | Reference | **0.027 (0.004, 0.050)** | **0.040 (0.002, 0.078)** | 0.023 (-0.026, 0.071) |
|  | BMC | Reference | 0.032 (-0.428, 0.491) | 0.297 (-0.294, 0.889) | -0.331 (-1.020, 0.358) |
|  | Area | Reference | **-0.414 (-0.807, -0.021)** | -0.226 (-0.656, 0.205) | **-0.740 (-1.308, -0.172)** |
| Intertrochanter | BMD | Reference | **0.049 (0.013, 0.085)** | 0.048 (-0.022, 0.117) | -0.009 (-0.075, 0.058) |
|  | BMC | Reference | **1.688 (0.777, 2.599)** | 0.931 (-1.430, 3.291) | 0.166 (-1.252, 1.585) |
|  | Area | Reference | 0.490 (-0.108, 1.088) | 0.008 (-1.137, 1.152) | 0.433 (-0.810, 1.676) |
| Wards triangle | BMD | Reference | 0.036 (-0.001, 0.074) | -0.005 (-0.068, 0.059) | -0.010 (-0.077, 0.058) |
|  | BMC | Reference | 0.039 (-0.010, 0.089) | -0.005 (-0.087, 0.077) | -0.019 (-0.098, 0.059) |
|  | Area | Reference | -0.003 (-0.022, 0.017) | -0.002 (-0.020, 0.015) | -0.011 (-0.042, 0.020) |

Note: Boldface type indicates statistical significance. An asterisk indicates that the product term of the interaction is significant.

Abbreviations: MASLD: metabolic dysfunction-associated steatotic liver disease; MetALD: metabolic alcohol-related liver disease; ALD: alcoholic liver disease; TG: triglyceride; BMD: bone mineral density; BMC: bone mineral content.

Supplementary table 9. Association of SLD subcategories with femur BMD, BMC and bone area stratified by HDL-c status.

|  |  | Normal | MASLD | MetALD | ALD |
| --- | --- | --- | --- | --- | --- |
|  |  |  | β (95% CI) | β (95% CI) | β (95% CI) |
| **Normal HDL** |  |  |  |  |  |
| Total femur | BMD | Reference | **0.053 (0.030, 0.077)** | **0.057 (0.013, 0.101)** | 0.027 (-0.012, 0.067) |
|  | BMC | Reference | **2.300 (1.112, 3.488)** | **2.186 (0.016, 4.356)** | 1.134 (-0.761, 3.028)* |
|  | Area | Reference | 0.126 (-0.418, 0.670) | 0.087 (-1.001, 1.174) | 0.165 (-1.259, 1.589) |
| Femur neck | BMD | Reference | **0.033 (0.007, 0.058)** | 0.035 (-0.008, 0.078) | 0.018 (-0.030, 0.065) |
|  | BMC | Reference | **0.177 (0.023, 0.331)** | 0.206 (-0.062, 0.474) | 0.036 (-0.205, 0.276)* |
|  | Area | Reference | -0.001 (-0.059, 0.057) | 0.044 (-0.046, 0.134) | -0.051 (-0.209, 0.108)* |
| Trochanter | BMD | Reference | **0.037 (0.016, 0.058)** | **0.042 (0.008, 0.076)** | **0.038 (0.005, 0.070)** |
|  | BMC | Reference | 0.315 (-0.034, 0.664) | **0.525 (0.022, 1.027)** | 0.060 (-0.523, 0.644)* |
|  | Area | Reference | -0.224 (-0.516, 0.068) | 0.031 (-0.325, 0.386) | -0.526 (-1.077, 0.024) |
| Intertrochanter | BMD | Reference | **0.062 (0.034, 0.090)** | **0.071 (0.019, 0.123)** | 0.012 (-0.037, 0.062)* |
|  | BMC | Reference | **1.808 (1.039, 2.576)** | 1.456 (-0.091, 3.003) | 1.037 (-0.177, 2.252)* |
|  | Area | Reference | 0.352 (-0.027, 0.730) | 0.013 (-0.747, 0.774) | 0.744 (-0.103, 1.590) |
| Wards triangle | BMD | Reference | **0.034 (0.007, 0.061)** | 0.010 (-0.050, 0.070) | -0.006 (-0.063, 0.051) |
|  | BMC | Reference | **0.041 (0.006, 0.075)** | 0.009 (-0.065, 0.083) | -0.015 (-0.088, 0.058) |
|  | Area | Reference | 0.002 (-0.011, 0.015) | -0.005 (-0.028, 0.017) | -0.008 (-0.033, 0.018) |
| **Abnormal HDL** |  |  |  |  |  |
| Total femur | BMD | Reference | **0.039 (0.014, 0.064)** | 0.045 (-0.051, 0.141) | **0.113 (0.021, 0.205)** |
|  | BMC | Reference | 1.227 (-0.147, 2.602) | 0.241 (-4.203, 4.684) | **6.079 (2.165, 9.994)** |
|  | Area | Reference | -0.140 (-0.906, 0.625) | -1.258 (-2.790, 0.275) | 2.149 (-0.169, 4.466) |
| Femur neck | BMD | Reference | 0.023 (-0.000, 0.046) | 0.055 (-0.015, 0.124) | **0.093 (0.021, 0.165)** |
|  | BMC | Reference | **0.160 (0.015, 0.305)** | 0.221 (-0.210, 0.653) | **0.655 (0.214, 1.097)** |
|  | Area | Reference | **0.067 (0.009, 0.125)** | -0.057 (-0.263, 0.149) | **0.223 (0.013, 0.433)** |
| Trochanter | BMD | Reference | **0.030 (0.010, 0.049)** | 0.054 (-0.018, 0.127) | **0.082 (0.000, 0.163)** |
|  | BMC | Reference | **0.415 (0.019, 0.811)** | 0.207 (-0.815, 1.230) | **1.229 (0.228, 2.230)** |
|  | Area | Reference | 0.120 (-0.211, 0.452) | -0.486 (-1.143, 0.172) | 0.545 (-0.475, 1.566) |
| Intertrochanter | BMD | Reference | **0.055 (0.021, 0.089)** | 0.039 (-0.076, 0.153) | **0.133 (0.025, 0.240)** |
|  | BMC | Reference | 0.652 (-0.375, 1.678) | -0.186 (-3.284, 2.911) | **4.195 (1.498, 6.892)** |
|  | Area | Reference | -0.328 (-1.027, 0.370) | -0.717 (-1.767, 0.334) | 1.382 (-0.031, 2.795) |
| Wards triangle | BMD | Reference | 0.012 (-0.021, 0.046) | 0.029 (-0.093, 0.151) | 0.094 (-0.019, 0.206) |
|  | BMC | Reference | 0.017 (-0.027, 0.060) | 0.032 (-0.141, 0.205) | 0.122 (-0.031, 0.274) |
|  | Area | Reference | 0.003 (-0.024, 0.030) | -0.018 (-0.063, 0.027) | 0.012 (-0.032, 0.055) |

Note: Boldface type indicates statistical significance. An asterisk indicates that the product term of the interaction is significant.

Abbreviations: MASLD: metabolic dysfunction-associated steatotic liver disease; MetALD: metabolic alcohol-related liver disease; ALD: alcoholic liver disease; ‌HDL: high-density lipoprotein; BMD: bone mineral density; BMC: bone mineral content.

Supplementary table 10. Association of SLD subcategories with femur BMD, BMC and bone area stratified by LSM value.

|  |  | Normal | MASLD | MetALD | ALD |
| --- | --- | --- | --- | --- | --- |
|  |  |  | β (95% CI) | β (95% CI) | β (95% CI) |
| **LSM<11.7** |  |  |  |  |  |
| Total femur | BMD | Reference | **0.055 (0.037, 0.073)** | **0.059 (0.012, 0.106)** | **0.058 (0.027, 0.090)*** |
|  | BMC | Reference | **2.047 (1.024, 3.069)** | 1.935 (-0.125, 3.995) | **2.290 (0.400, 4.180)** |
|  | Area | Reference | -0.070 (-0.579, 0.438)* | -0.056 (-0.876, 0.764) | 0.169 (-1.403, 1.742) |
| Femur neck | BMD | Reference | **0.032 (0.012, 0.052)** | 0.041 (-0.000, 0.083) | **0.054 (0.020, 0.089)*** |
|  | BMC | Reference | **0.186 (0.063, 0.309)** | 0.229 (-0.012, 0.470) | **0.245 (0.057, 0.433)*** |
|  | Area | Reference | 0.025 (-0.028, 0.078) | 0.042 (-0.050, 0.135) | -0.038 (-0.193, 0.118) |
| Trochanter | BMD | Reference | **0.041 (0.025, 0.058)** | **0.048 (0.014, 0.083)** | **0.057 (0.028, 0.087)*** |
|  | BMC | Reference | **0.389 (0.105, 0.674)** | **0.548 (0.118, 0.978)** | 0.456 (-0.065, 0.978) |
|  | Area | Reference | -0.150 (-0.384, 0.085) | 0.036 (-0.237, 0.310) | -0.277 (-0.887, 0.333) |
| Intertrochanter | BMD | Reference | **0.067 (0.047, 0.087)** | **0.070 (0.014, 0.127)** | **0.053 (0.015, 0.092)*** |
|  | BMC | Reference | **1.471 (0.770, 2.173)** | 1.159 (-0.339, 2.657) | **1.588 (0.272, 2.903)** |
|  | Area | Reference | 0.055 (-0.320, 0.430) | -0.134 (-0.761, 0.492) | 0.485 (-0.429, 1.400)* |
| Wards triangle | BMD | Reference | **0.033 (0.011, 0.056)** | 0.017 (-0.045, 0.080) | 0.039 (-0.005, 0.083)* |
|  | BMC | Reference | **0.040 (0.011, 0.068)** | 0.016 (-0.067, 0.099) | 0.038 (-0.020, 0.096)* |
|  | Area | Reference | 0.002 (-0.010, 0.015) | -0.011 (-0.034, 0.012) | -0.008 (-0.029, 0.013) |
| **LSM≥11.7** |  |  |  |  |  |
| Total femur | BMD | Reference | 0.009 (-0.121, 0.139) | 0.036 (-0.137, 0.209) | -0.135 (-0.293, 0.022) |
|  | BMC | Reference | 4.102 (-1.877, 10.081) | 1.964 (-4.038, 7.966) | -2.444 (-9.074, 4.185) |
|  | Area | Reference | **3.000 (1.115, 4.886)** | -0.803 (-3.065, 1.458) | 2.489 (-1.444, 6.421) |
| Femur neck | BMD | Reference | 0.015 (-0.081, 0.110) | 0.025 (-0.103, 0.153) | **-0.176 (-0.292, -0.059)** |
|  | BMC | Reference | 0.235 (-0.173, 0.642) | 0.203 (-0.458, 0.865) | **-0.742 (-1.337, -0.148)** |
|  | Area | Reference | 0.098 (-0.176, 0.372) | -0.081 (-0.599, 0.438) | 0.172 (-0.216, 0.561) |
| Trochanter | BMD | Reference | -0.012 (-0.137, 0.112) | 0.028 (-0.122, 0.179) | -0.092 (-0.226, 0.043) |
|  | BMC | Reference | 0.498 (-0.786, 1.781) | 0.063 (-1.596, 1.721) | -1.014 (-2.895, 0.866) |
|  | Area | Reference | 0.545 (-0.489, 1.580) | -1.018 (-2.421, 0.384) | -0.020 (-1.789, 1.749) |
| Intertrochanter | BMD | Reference | 0.011 (-0.114, 0.137) | 0.024 (-0.173, 0.222) | -0.168 (-0.352, 0.017) |
|  | BMC | Reference | 3.371 (-1.107, 7.849) | 1.703 (-2.497, 5.903) | -0.680 (-5.135, 3.775) |
|  | Area | Reference | **2.354 (0.336, 4.372)** | 0.291 (-1.337, 1.919) | 2.334 (-0.022, 4.689) |
| Wards triangle | BMD | Reference | -0.030 (-0.152, 0.091) | -0.023 (-0.184, 0.138) | -0.227 (-0.353, -0.102) |
|  | BMC | Reference | -0.046 (-0.225, 0.132) | -0.020 (-0.247, 0.207) | -0.298 (-0.481, -0.115) |
|  | Area | Reference | -0.009 (-0.066, 0.047) | 0.019 (-0.052, 0.090) | -0.029 (-0.101, 0.043) |

Note: Boldface type indicates statistical significance. An asterisk indicates that the product term of the interaction is significant.

Abbreviations: MASLD: metabolic dysfunction-associated steatotic liver disease; MetALD: metabolic alcohol-related liver disease; ALD: alcoholic liver disease; ‌ LSM: liver stiffness measurement; BMD: bone mineral density; BMC: bone mineral content.

Supplementary table 11. Association of SLD subcategories with spine BMD, BMC and bone area stratified by age.

|  | | Normal | MASLD  β (95% CI) | MetALD  β (95% CI) | ALD  β (95% CI) |
| --- | --- | --- | --- | --- | --- |
| **Age<65** |  |  |  |  |  |
| Total spine | BMD | Reference | **0.064 (0.031, 0.097)** | **0.059 (0.019, 0.099)** | **0.080 (0.025, 0.136)*** |
|  | BMC | Reference | **3.057 (0.807, 5.307)** | 2.831 (-0.841, 6.503) | **7.023 (1.806, 12.239)** |
|  | Area | Reference | -0.803 (-2.048, 0.441) | -0.534 (-2.479, 1.412) | 1.752 (-0.751, 4.256) |
| L1 | BMD | Reference | **0.075 (0.045, 0.104)** | **0.052 (0.019, 0.085)** | **0.066 (0.016, 0.116)*** |
|  | BMC | Reference | **1.055 (0.448, 1.661)** | 0.660 (-0.057, 1.376) | 0.961 (-0.079, 2.001)* |
|  | Area | Reference | 0.068 (-0.182, 0.319) | 0.054 (-0.423, 0.530) | 0.186 (-0.361, 0.732)* |
| L2 | BMD | Reference | **0.063 (0.030, 0.096)** | **0.060 (0.017, 0.104)** | **0.091 (0.043, 0.139)** |
|  | BMC | Reference | **0.796 (0.075, 1.516)** | 0.851 (-0.158, 1.860) | **1.789 (0.706, 2.871)** |
|  | Area | Reference | -0.158 (-0.525, 0.209) | -0.009 (-0.550, 0.532) | 0.373 (-0.185, 0.930) |
| L3 | BMD | Reference | **0.065 (0.028, 0.103)** | **0.068 (0.025, 0.112)** | **0.095 (0.035, 0.156)** |
|  | BMC | Reference | 0.757 (-0.218, 1.733) | 0.916 (-0.241, 2.073) | **1.783 (0.299, 3.267)** |
|  | Area | Reference | -0.304 (-0.806, 0.199) | -0.135 (-0.689, 0.419) | 0.161 (-0.458, 0.779) |
| L4 | BMD | Reference | **0.059 (0.020, 0.097)** | **0.063 (0.011, 0.116)** | 0.068 (-0.002, 0.138) |
|  | BMC | Reference | **0.913 (0.109, 1.717)** | 0.456 (-0.992, 1.904) | **1.941 (0.154, 3.729)** |
|  | Area | Reference | -0.135 (-0.431, 0.161) | -0.514 (-1.222, 0.194) | 0.695 (-0.245, 1.636) |
| **Age≥65** |  |  |  |  |  |
| Total spine | BMD | Reference | **0.050 (0.004, 0.095)** | **0.111 (0.025, 0.198)** | -0.002 (-0.060, 0.057) |
|  | BMC | Reference | 2.579 (-1.886, 7.044) | 3.246 (-2.878, 9.370) | -1.736 (-10.723, 7.251) |
|  | Area | Reference | -0.209 (-3.033, 2.614) | -2.200 (-5.461, 1.061) | -0.726 (-5.771, 4.320) |
| L1 | BMD | Reference | **0.044 (0.000, 0.087)** | **0.112 (0.038, 0.185)** | -0.022 (-0.080, 0.036) |
|  | BMC | Reference | 0.541 (-0.383, 1.466) | 0.877 (-0.718, 2.472) | -1.672 (-3.380, 0.037) |
|  | Area | Reference | -0.043 (-0.560, 0.474) | -0.557 (-1.278, 0.163) | **-1.181 (-2.158, -0.204)** |
| L2 | BMD | Reference | 0.039 (-0.013, 0.090) | **0.114 (0.021, 0.208)** | 0.023 (-0.044, 0.091) |
|  | BMC | Reference | 0.486 (-0.559, 1.532) | 1.343 (-0.707, 3.393) | -0.248 (-2.689, 2.194) |
|  | Area | Reference | -0.064 (-0.472, 0.345) | -0.248 (-1.160, 0.664) | -0.446 (-1.707, 0.815) |
| L3 | BMD | Reference | **0.057 (0.008, 0.106)** | **0.119 (0.020, 0.217)** | 0.012 (-0.050, 0.075) |
|  | BMC | Reference | 0.985 (-0.139, 2.108) | 1.799 (-0.539, 4.136) | 0.361 (-1.997, 2.719) |
|  | Area | Reference | 0.092 (-0.647, 0.831) | -0.068 (-1.075, 0.939) | 0.253 (-1.082, 1.587) |
| L4 | BMD | Reference | **0.065 (0.017, 0.112)** | 0.089 (-0.001, 0.180) | -0.021 (-0.084, 0.042) |
|  | BMC | Reference | 1.387 (-0.023, 2.797) | 0.588 (-1.355, 2.530) | 0.491 (-3.150, 4.133) |
|  | Area | Reference | 0.291 (-0.593, 1.176) | -0.759 (-1.754, 0.235) | 1.098 (-1.378, 3.575) |

Note: Boldface type indicates statistical significance. An asterisk indicates that the product term of the interaction is significant.

Abbreviations: MASLD: metabolic dysfunction-associated steatotic liver disease; MetALD: metabolic alcohol-related liver disease; ALD: alcoholic liver disease; BMD: bone mineral density; BMC: bone mineral content.

Supplementary table 12. Association of SLD subcategories with spine BMD, BMC and bone area stratified by gender.

|  |  | Normal | MASLD | MetALD | ALD |
| --- | --- | --- | --- | --- | --- |
|  |  |  | β (95% CI) | β (95% CI) | β (95% CI) |
| **Male** |  |  |  |  |  |
| Total spine | BMD | Reference | **0.057 (0.013, 0.100)** | -0.016 (-0.086, 0.054)* | **0.052 (0.006, 0.099)** |
|  | BMC | Reference | 1.920 (-2.069, 5.909) | **-5.498 (-10.418, -0.577)*** | 2.979 (-1.684, 7.643) |
|  | Area | Reference | **-1.852 (-3.407, -0.297)** | **-3.900 (-5.692, -2.108)*** | -0.497 (-2.809, 1.814) |
| L1 | BMD | Reference | **0.059 (0.024, 0.094)** | -0.025 (-0.085, 0.035)* | 0.025 (-0.028, 0.077) |
|  | BMC | Reference | 0.660 (-0.059, 1.380) | **-1.336 (-2.411, -0.260)*** | -0.383 (-1.913, 1.146)* |
|  | Area | Reference | -0.225 (-0.609, 0.159) | **-0.940 (-1.405, -0.476)*** | -0.699 (-1.531, 0.133)* |
| L2 | BMD | Reference | **0.054 (0.008, 0.099)** | -0.011 (-0.079, 0.058)* | **0.063 (0.011, 0.116)** |
|  | BMC | Reference | 0.607 (-0.529, 1.743) | -0.732 (-2.223, 0.758)* | 0.921 (-0.405, 2.247) |
|  | Area | Reference | -0.300 (-0.784, 0.184) | -0.591 (-1.213, 0.031)* | -0.112 (-0.670, 0.447) |
| L3 | BMD | Reference | **0.062 (0.013, 0.111)** | -0.012 (-0.088, 0.064)* | **0.069 (0.016, 0.123)** |
|  | BMC | Reference | 0.665 (-0.607, 1.938) | -1.083 (-2.672, 0.506)* | 0.886 (-0.207, 1.980) |
|  | Area | Reference | -0.451 (-0.950, 0.047) | **-0.887 (-1.222, -0.553)*** | -0.340 (-0.736, 0.055) |
| L4 | BMD | Reference | **0.061 (0.011, 0.111)** | -0.020 (-0.103, 0.062)* | 0.043 (-0.010, 0.095) |
|  | BMC | Reference | 0.739 (-0.393, 1.870) | -1.474 (-3.365, 0.417)* | 0.938 (-0.617, 2.493) |
|  | Area | Reference | -0.432 (-0.982, 0.117)* | **-1.070 (-1.865, -0.275)** | 0.166 (-0.928, 1.260) |
| **Female** |  |  |  |  |  |
| Total spine | BMD | Reference | **0.059 (0.010, 0.107)** | **0.099 (0.066, 0.133)** | **0.138 (0.013, 0.262)** |
|  | BMC | Reference | **3.558 (0.038, 7.078)** | **6.880 (3.421, 10.338)** | **10.765 (0.858, 20.672)** |
|  | Area | Reference | 0.556 (-2.831, 3.943) | 1.154 (-1.182, 3.491) | 2.267 (-1.778, 6.312) |
| L1 | BMD | Reference | **0.061 (0.017, 0.105)** | **0.091 (0.066, 0.116)** | **0.136 (0.019, 0.253)** |
|  | BMC | Reference | **0.933 (0.178, 1.687)** | **1.558 (1.013, 2.102)** | **2.304 (0.401, 4.208)** |
|  | Area | Reference | 0.193 (-0.222, 0.608) | 0.359 (-0.129, 0.848) | 0.568 (-0.217, 1.353) |
| L2 | BMD | Reference | **0.053 (0.005, 0.101)** | **0.098 (0.064, 0.133)** | **0.158 (0.041, 0.275)** |
|  | BMC | Reference | 0.683 (-0.069, 1.434) | **1.693 (0.829, 2.557)** | **2.850 (0.441, 5.259)** |
|  | Area | Reference | 0.015 (-0.509, 0.540) | 0.303 (-0.255, 0.860) | 0.584 (-0.402, 1.571) |
| L3 | BMD | Reference | **0.062 (0.006, 0.119)** | **0.112 (0.073, 0.151)** | **0.155 (0.043, 0.267)** |
|  | BMC | Reference | **0.984 (0.056, 1.911)** | **2.254 (1.034, 3.475)** | **3.128 (0.448, 5.807)** |
|  | Area | Reference | 0.117 (-0.572, 0.805) | 0.513 (-0.167, 1.193) | 0.689 (-0.440, 1.818) |
| L4 | BMD | Reference | **0.059 (0.010, 0.109)** | **0.100 (0.053, 0.146)** | 0.121 (-0.028, 0.269) |
|  | BMC | Reference | **1.416 (0.474, 2.359)** | **1.430 (0.032, 2.829)** | **3.440 (0.388, 6.492)** |
|  | Area | Reference | 0.494 (-0.250, 1.238) | -0.107 (-0.962, 0.749) | **1.368 (0.614, 2.123)** |

Note: Boldface type indicates statistical significance. An asterisk indicates that the product term of the interaction is significant.

Abbreviations: MASLD: metabolic dysfunction-associated steatotic liver disease; MetALD: metabolic alcohol-related liver disease; ALD: alcoholic liver disease; BMD: bone mineral density; BMC: bone mineral content.

Supplementary table 13. Association of SLD subcategories with spine BMD, BMC and bone area stratified by BMI.

|  |  | Normal | MASLD | MetALD | ALD |
| --- | --- | --- | --- | --- | --- |
|  |  |  | β (95% CI) | β (95% CI) | β (95% CI) |
| **BMI<25** |  |  |  |  |  |
| Total spine | BMD | Reference | **0.081 (0.006, 0.156)** | **-0.065 (-0.109, -0.021)*** | **0.220 (0.145, 0.294)*** |
|  | BMC | Reference | **7.189 (0.471, 13.907)** | **-5.742 (-10.098, -1.386)*** | **12.779 (6.280, 19.279)*** |
|  | Area | Reference | 1.492 (-1.621, 4.605) | -1.150 (-3.444, 1.143) | -3.193 (-7.263, 0.878) |
| L1 | BMD | Reference | 0.076 (-0.001, 0.153) | -0.043 (-0.102, 0.016)* | **0.211 (0.123, 0.298)*** |
|  | BMC | Reference | **1.808 (0.122, 3.495)** | -0.925 (-1.931, 0.080) | **5.057 (3.529, 6.586)*** |
|  | Area | Reference | 0.552 (-0.115, 1.218) | 0.166 (-0.682, 1.014) | 1.061 (-0.014, 2.136)* |
| L2 | BMD | Reference | 0.082 (-0.000, 0.164) | **-0.052 (-0.087, -0.017)*** | **0.189 (0.108, 0.270)*** |
|  | BMC | Reference | **1.935 (0.281, 3.588)** | -0.641 (-1.527, 0.244) | 1.031 (-0.597, 2.660) |
|  | Area | Reference | 0.511 (-0.065, 1.087)* | 0.189 (-0.633, 1.011) | **-1.893 (-2.764, -1.021)*** |
| L3 | BMD | Reference | **0.078 (0.001, 0.156)** | **-0.052 (-0.092, -0.013)*** | **0.266 (0.181, 0.351)*** |
|  | BMC | Reference | 1.491 (-0.289, 3.271) | **-1.685 (-2.634, -0.737)*** | **3.232 (1.440, 5.024)*** |
|  | Area | Reference | 0.067 (-0.663, 0.796) | **-0.774 (-1.307, -0.242)** | **-1.461 (-2.472, -0.451)*** |
| L4 | BMD | Reference | **0.079 (0.010, 0.148)** | **-0.098 (-0.167, -0.028)*** | **0.204 (0.130, 0.279)*** |
|  | BMC | Reference | 1.334 (-0.361, 3.028) | **-2.873 (-5.154, -0.591)*** | **3.361 (1.618, 5.104)** |
|  | Area | Reference | -0.240 (-1.426, 0.946) | -1.149 (-2.444, 0.147) | -0.808 (-1.971, 0.356)* |
| **BMI≥25** |  |  |  |  |  |
| Total spine | BMD | Reference | 0.030 (-0.010, 0.071) | **0.047 (0.018, 0.077)** | 0.032 (-0.020, 0.084) |
|  | BMC | Reference | 1.096 (-1.749, 3.941) | 1.836 (-1.996, 5.668) | 3.392 (-1.149, 7.933) |
|  | Area | Reference | -0.530 (-2.429, 1.369) | -0.612 (-2.728, 1.504) | 1.374 (-1.031, 3.780) |
| L1 | BMD | Reference | 0.032 (-0.004, 0.068) | **0.039 (0.014, 0.065)** | 0.010 (-0.042, 0.062) |
|  | BMC | Reference | 0.383 (-0.258, 1.024) | 0.364 (-0.487, 1.215) | -0.159 (-1.417, 1.099) |
|  | Area | Reference | -0.037 (-0.277, 0.203) | -0.118 (-0.671, 0.435) | -0.221 (-0.895, 0.453) |
| L2 | BMD | Reference | 0.026 (-0.017, 0.070) | **0.050 (0.017, 0.083)** | 0.047 (-0.001, 0.094) |
|  | BMC | Reference | 0.182 (-0.610, 0.974) | 0.644 (-0.338, 1.625) | 0.883 (-0.188, 1.954) |
|  | Area | Reference | -0.174 (-0.528, 0.179) | -0.062 (-0.577, 0.454) | 0.201 (-0.354, 0.755) |
| L3 | BMD | Reference | 0.035 (-0.011, 0.081) | **0.056 (0.022, 0.089)** | 0.048 (-0.005, 0.102) |
|  | BMC | Reference | 0.362 (-0.567, 1.290) | 0.849 (-0.298, 1.995) | 0.905 (-0.268, 2.078) |
|  | Area | Reference | -0.174 (-0.666, 0.318) | -0.015 (-0.632, 0.602) | 0.120 (-0.400, 0.641) |
| L4 | BMD | Reference | 0.032 (-0.010, 0.074) | **0.046 (0.011, 0.081)** | 0.016 (-0.050, 0.082) |
|  | BMC | Reference | 0.718 (-0.183, 1.619) | 0.332 (-0.905, 1.569) | 1.127 (-0.285, 2.540) |
|  | Area | Reference | 0.190 (-0.250, 0.630) | -0.323 (-0.995, 0.350) | 0.894 (-0.015, 1.803) |

Note: Boldface type indicates statistical significance. An asterisk indicates that the product term of the interaction is significant.

Abbreviations: MASLD: metabolic dysfunction-associated steatotic liver disease; MetALD: metabolic alcohol-related liver disease; ALD: alcoholic liver disease; BMI: body mass index; BMD: bone mineral density; BMC: bone mineral content.

Supplementary table 14. Association of SLD subcategories with spine BMD, BMC and bone area stratified by glucose status.

|  |  | Normal | MASLD | MetALD | ALD |
| --- | --- | --- | --- | --- | --- |
|  |  |  | β (95% CI) | β (95% CI) | β (95% CI) |
| **Normal glucose** |  |  |  |  |  |
| Total spine | BMD | Reference | 0.028 (-0.023, 0.079) | **0.068 (0.011, 0.125)** | 0.163 (-0.025, 0.352) |
|  | BMC | Reference | 1.105 (-1.815, 4.026) | **5.252 (0.110, 10.393)** | 12.003 (-1.736, 25.742) |
|  | Area | Reference | -0.008 (-3.491, 3.475) | 1.301 (-1.338, 3.941) | 2.090 (-0.785, 4.965) |
| L1 | BMD | Reference | 0.036 (-0.015, 0.088) | **0.059 (0.001, 0.118)** | 0.144 (-0.035, 0.323) |
|  | BMC | Reference | 0.331 (-0.277, 0.939) | 0.713 (-0.106, 1.532) | 1.625 (-1.191, 4.441) |
|  | Area | Reference | 0.067 (-0.596, 0.730) | 0.021 (-0.488, 0.529) | -0.125 (-0.958, 0.708) |
| L2 | BMD | Reference | 0.025 (-0.032, 0.082) | **0.080 (0.016, 0.143)** | 0.158 (-0.032, 0.347) |
|  | BMC | Reference | 0.213 (-0.473, 0.900) | **1.422 (0.124, 2.720)** | 2.609 (-0.783, 6.000) |
|  | Area | Reference | -0.042 (-0.638, 0.555) | 0.294 (-0.276, 0.864) | 0.343 (-0.290, 0.976) |
| L3 | BMD | Reference | 0.039 (-0.015, 0.093) | **0.071 (0.005, 0.138)** | 0.159 (-0.022, 0.341) |
|  | BMC | Reference | 0.311 (-0.437, 1.058) | **1.795 (0.123, 3.467)** | 2.689 (-0.995, 6.373) |
|  | Area | Reference | -0.201 (-0.969, 0.568) | 0.628 (-0.143, 1.400)* | 0.163 (-0.915, 1.242) |
| L4 | BMD | Reference | 0.046 (-0.007, 0.098) | **0.065 (0.012, 0.119)** | 0.182 (-0.031, 0.396) |
|  | BMC | Reference | 1.041 (-0.239, 2.320) | 1.250 (-0.634, 3.134) | 4.576 (-0.265, 9.417) |
|  | Area | Reference | 0.370 (-1.159, 1.898) | 0.129 (-1.064, 1.322) | 1.300 (-0.149, 2.750) |
| **Abnormal glucose** |  |  |  |  |  |
| Total spine | BMD | Reference | **0.061 (0.020, 0.103)** | **0.072 (0.017, 0.127)** | **0.048 (0.007, 0.090)** |
|  | BMC | Reference | 3.133 (-0.427, 6.692) | 1.716 (-2.774, 6.206) | 3.805 (-0.655, 8.266) |
|  | Area | Reference | -0.533 (-2.148, 1.081) | -2.012 (-4.483, 0.460) | 0.724 (-1.904, 3.352) |
| L1 | BMD | Reference | **0.062 (0.024, 0.100)** | **0.065 (0.018, 0.111)** | 0.023 (-0.023, 0.068) |
|  | BMC | Reference | **0.904 (0.179, 1.630)** | 0.728 (-0.454, 1.909) | 0.059 (-1.304, 1.422) |
|  | Area | Reference | 0.050 (-0.244, 0.343) | -0.114 (-0.808, 0.580) | -0.213 (-0.970, 0.543) |
| L2 | BMD | Reference | **0.055 (0.011, 0.098)** | **0.066 (0.010, 0.123)** | **0.063 (0.025, 0.100)** |
|  | BMC | Reference | 0.661 (-0.234, 1.556) | 0.692 (-0.480, 1.863) | **1.072 (0.054, 2.089)** |
|  | Area | Reference | -0.165 (-0.500, 0.171) | -0.249 (-0.841, 0.342) | 0.128 (-0.435, 0.690) |
| L3 | BMD | Reference | **0.063 (0.019, 0.107)** | **0.086 (0.025, 0.147)** | **0.070 (0.024, 0.116)** |
|  | BMC | Reference | 0.881 (-0.167, 1.929) | 0.782 (-0.578, 2.142) | **1.165 (0.009, 2.321)** |
|  | Area | Reference | -0.134 (-0.622, 0.354) | **-0.517 (-0.935, -0.098)** | 0.030 (-0.543, 0.604) |
| L4 | BMD | Reference | **0.062 (0.017, 0.107)** | **0.072 (0.007, 0.137)** | 0.035 (-0.017, 0.086) |
|  | BMC | Reference | **1.091 (0.082, 2.099)** | 0.136 (-1.181, 1.453) | 1.079 (-0.173, 2.332) |
|  | Area | Reference | -0.006 (-0.381, 0.369) | **-0.882 (-1.482, -0.283)** | 0.535 (-0.448, 1.518) |

Note: Boldface type indicates statistical significance. An asterisk indicates that the product term of the interaction is significant.

Abbreviations: MASLD: metabolic dysfunction-associated steatotic liver disease; MetALD: metabolic alcohol-related liver disease; ALD: alcoholic liver disease; BMD: bone mineral density; BMC: bone mineral content.

Supplementary table 15. Association of SLD subcategories with spine BMD, BMC and bone area stratified by TG status.

|  |  | Normal | MASLD | MetALD | ALD |
| --- | --- | --- | --- | --- | --- |
|  |  |  | β (95% CI) | β (95% CI) | β (95% CI) |
| **Normal TG** |  |  |  |  |  |
| Total spine | BMD | Reference | 0.034 (-0.007, 0.075) | 0.056 (-0.013, 0.125) | **0.094 (0.021, 0.167)** |
|  | BMC | Reference | 0.677 (-2.376, 3.730) | 1.790 (-2.667, 6.246) | **7.925 (1.463, 14.387)** |
|  | Area | Reference | -1.077 (-4.678, 2.524) | -1.080 (-4.360, 2.200) | 2.033 (-0.505, 4.572) |
| L1 | BMD | Reference | **0.043 (0.003, 0.083)** | 0.051 (-0.009, 0.112) | **0.075 (0.004, 0.146)** |
|  | BMC | Reference | 0.441 (-0.190, 1.071) | 0.316 (-0.682, 1.315) | 1.077 (-0.502, 2.656) |
|  | Area | Reference | -0.081 (-0.560, 0.398) | -0.218 (-0.851, 0.415) | 0.168 (-0.597, 0.933) |
| L2 | BMD | Reference | 0.028 (-0.017, 0.073) | **0.067 (0.001, 0.134)** | **0.099 (0.023, 0.175)** |
|  | BMC | Reference | 0.164 (-0.496, 0.824) | 0.916 (-0.322, 2.155) | **1.729 (0.044, 3.414)** |
|  | Area | Reference | -0.211 (-0.756, 0.335) | 0.004 (-0.682, 0.690) | 0.261 (-0.359, 0.880) |
| L3 | BMD | Reference | 0.038 (-0.012, 0.089) | 0.071 (-0.007, 0.149) | **0.102 (0.027, 0.177)** |
|  | BMC | Reference | 0.335 (-0.432, 1.101) | 1.037 (-0.593, 2.666) | **1.676 (0.003, 3.348)** |
|  | Area | Reference | -0.219 (-0.925, 0.487) | -0.019 (-0.712, 0.673) | 0.021 (-0.597, 0.638) |
| L4 | BMD | Reference | **0.041 (0.003, 0.080)** | 0.049 (-0.052, 0.151) | 0.091 (-0.005, 0.187) |
|  | BMC | Reference | **0.756 (0.099, 1.413)** | 0.091 (-1.742, 1.924) | **2.451 (0.157, 4.746)** |
|  | Area | Reference | 0.104 (-0.655, 0.863) | -0.559 (-1.555, 0.436) | 0.814 (-0.201, 1.828) |
| **Abnormal TG** |  |  |  |  |  |
| Total spine | BMD | Reference | **0.069 (0.030, 0.109)** | **0.074 (0.015, 0.133)** | 0.032 (-0.022, 0.086) |
|  | BMC | Reference | **4.398 (0.569, 8.227)** | 2.920 (-1.660, 7.499) | 3.092 (-2.476, 8.659) |
|  | Area | Reference | 0.327 (-1.548, 2.201) | -0.972 (-3.102, 1.158) | 1.287 (-2.342, 4.916) |
| L1 | BMD | Reference | **0.066 (0.033, 0.100)** | **0.059 (0.011, 0.106)** | 0.006 (-0.047, 0.059) |
|  | BMC | Reference | **0.988 (0.019, 1.956)** | 0.669 (-0.551, 1.889) | -0.406 (-1.730, 0.919) |
|  | Area | Reference | 0.096 (-0.478, 0.669) | -0.110 (-0.700, 0.480) | -0.397 (-1.139, 0.345) |
| L2 | BMD | Reference | **0.066 (0.025, 0.107)** | **0.063 (0.007, 0.119)** | 0.048 (-0.003, 0.099) |
|  | BMC | Reference | 0.861 (-0.199, 1.920) | 0.711 (-0.636, 2.058) | 0.998 (-0.405, 2.401) |
|  | Area | Reference | -0.066 (-0.546, 0.414) | -0.194 (-0.759, 0.371) | 0.347 (-0.483, 1.177) |
| L3 | BMD | Reference | **0.072 (0.027, 0.117)** | **0.079 (0.011, 0.147)** | 0.054 (-0.003, 0.111) |
|  | BMC | Reference | 0.975 (-0.132, 2.082) | 0.969 (-0.493, 2.430) | 1.218 (-0.143, 2.580) |
|  | Area | Reference | -0.145 (-0.776, 0.487) | -0.237 (-0.906, 0.432) | 0.373 (-0.399, 1.145) |
| L4 | BMD | Reference | **0.061 (0.017, 0.105)** | **0.076 (0.004, 0.148)** | 0.013 (-0.045, 0.071) |
|  | BMC | Reference | **1.022 (0.031, 2.012)** | 0.519 (-0.792, 1.831) | 0.957 (-0.533, 2.447) |
|  | Area | Reference | -0.021 (-0.472, 0.431) | -0.664 (-1.357, 0.030) | 0.823 (-0.473, 2.120) |

Note: Boldface type indicates statistical significance. An asterisk indicates that the product term of the interaction is significant.

Abbreviations: MASLD: metabolic dysfunction-associated steatotic liver disease; MetALD: metabolic alcohol-related liver disease; ALD: alcoholic liver disease; TG: triglyceride; BMD: bone mineral density; BMC: bone mineral content.

Supplementary table 16. Association of SLD subcategories with spine BMD, BMC and bone area stratified by HDL-c status.

|  |  | Normal | MASLD | MetALD | ALD |
| --- | --- | --- | --- | --- | --- |
|  |  |  | β (95% CI) | β (95% CI) | β (95% CI) |
| **Normal HDL** |  |  |  |  |  |
| Total spine | BMD | Reference | **0.059 (0.028, 0.090)** | **0.062 (0.019, 0.106)** | 0.031 (-0.005, 0.068)* |
|  | BMC | Reference | **3.167 (0.513, 5.821)*** | 2.997 (-1.047, 7.042) | 2.975 (-0.563, 6.513)* |
|  | Area | Reference | -0.643 (-1.632, 0.345) | -0.635 (-2.459, 1.189) | 0.878 (-1.188, 2.945) |
| L1 | BMD | Reference | **0.059 (0.030, 0.088)** | **0.059 (0.020, 0.097)** | 0.008 (-0.027, 0.044)* |
|  | BMC | Reference | **0.833 (0.231, 1.435)** | 0.628 (-0.372, 1.628) | -0.291 (-1.294, 0.712)* |
|  | Area | Reference | -0.063 (-0.387, 0.261) | -0.215 (-0.679, 0.249) | -0.401 (-1.008, 0.205) |
| L2 | BMD | Reference | **0.053 (0.020, 0.086)** | **0.060 (0.013, 0.107)** | **0.045 (0.006, 0.084)*** |
|  | BMC | Reference | 0.664 (-0.023, 1.352) | 0.770 (-0.436, 1.977) | 0.871 (-0.116, 1.859)* |
|  | Area | Reference | -0.157 (-0.417, 0.103) | -0.152 (-0.676, 0.372) | 0.167 (-0.346, 0.679) |
| L3 | BMD | Reference | **0.061 (0.025, 0.097)** | **0.067 (0.019, 0.116)** | **0.051 (0.008, 0.093)*** |
|  | BMC | Reference | 0.783 (-0.101, 1.668) | 0.981 (-0.280, 2.242) | 0.828 (-0.172, 1.829)* |
|  | Area | Reference | -0.241 (-0.653, 0.171) | -0.145 (-0.743, 0.454) | -0.036 (-0.498, 0.426)* |
| L4 | BMD | Reference | **0.064 (0.032, 0.097)** | **0.052 (0.002, 0.103)** | 0.016 (-0.027, 0.060)* |
|  | BMC | Reference | **1.195 (0.428, 1.961)** | 0.477 (-0.689, 1.644) | **1.205 (0.211, 2.198)** |
|  | Area | Reference | -0.017 (-0.407, 0.373) | -0.417 (-1.059, 0.225) | **0.897 (0.053, 1.740)** |
| **Abnormal HDL** |  |  |  |  |  |
| Total spine | BMD | Reference | 0.008 (-0.069, 0.086) | 0.078 (-0.027, 0.183) | **0.248 (0.068, 0.428)** |
|  | BMC | Reference | -1.237 (-4.141, 1.668) | 1.890 (-3.973, 7.754) | **20.404 (6.362, 34.445)** |
|  | Area | Reference | -0.481 (-5.849, 4.888) | -1.985 (-5.187, 1.217) | **4.758 (1.042, 8.473)** |
| L1 | BMD | Reference | 0.022 (-0.054, 0.098) | 0.064 (-0.031, 0.159) | **0.230 (0.062, 0.398)** |
|  | BMC | Reference | 0.152 (-0.540, 0.844) | 0.561 (-0.761, 1.882) | **4.207 (0.990, 7.424)** |
|  | Area | Reference | 0.118 (-0.578, 0.815) | 0.003 (-0.685, 0.692) | 1.071 (-0.304, 2.445) |
| L2 | BMD | Reference | 0.001 (-0.086, 0.088) | 0.089 (-0.006, 0.183) | **0.243 (0.062, 0.423)** |
|  | BMC | Reference | -0.268 (-1.275, 0.739) | 1.175 (-0.317, 2.667) | **4.431 (1.073, 7.789)** |
|  | Area | Reference | -0.123 (-0.904, 0.659) | -0.015 (-0.601, 0.570) | **0.879 (0.178, 1.581)** |
| L3 | BMD | Reference | 0.015 (-0.067, 0.097) | 0.103 (-0.009, 0.215) | **0.244 (0.064, 0.425)** |
|  | BMC | Reference | -0.160 (-1.110, 0.790) | 1.391 (-1.016, 3.798) | **5.009 (1.523, 8.495)** |
|  | Area | Reference | -0.190 (-1.307, 0.927) | -0.116 (-0.997, 0.765) | **1.043 (0.316, 1.770)** |
| L4 | BMD | Reference | 0.005 (-0.065, 0.076) | 0.085 (-0.067, 0.238) | **0.248 (0.033, 0.463)** |
|  | BMC | Reference | 0.306 (-0.782, 1.393) | 0.152 (-2.655, 2.959) | **5.945 (0.645, 11.244)** |
|  | Area | Reference | 0.461 (-1.096, 2.018) | -0.985 (-2.075, 0.106) | 1.344 (-0.112, 2.799) |

Note: Boldface type indicates statistical significance. An asterisk indicates that the product term of the interaction is significant.

Abbreviations: MASLD: metabolic dysfunction-associated steatotic liver disease; MetALD: metabolic alcohol-related liver disease; ALD: alcoholic liver disease; ‌HDL: high-density lipoprotein; BMD: bone mineral density; BMC: bone mineral content.

Supplementary table 17. Association of SLD subcategories with spine BMD, BMC and bone area stratified by LSM value.

|  |  | Normal | MASLD | MetALD | ALD |
| --- | --- | --- | --- | --- | --- |
|  |  |  | β (95% CI) | β (95% CI) | β (95% CI) |
| **LSM<11.7** |  |  |  |  |  |
| Total spine | BMD | Reference | **0.055 (0.023, 0.087)** | **0.069 (0.032, 0.107)** | **0.093 (0.032, 0.154)*** |
|  | BMC | Reference | **3.046 (0.917, 5.175)** | 3.286 (-0.228, 6.799) | **7.325 (1.896, 12.755)** |
|  | Area | Reference | -0.100 (-1.551, 1.351) | -0.392 (-2.383, 1.600) | 1.418 (-1.206, 4.041) |
| L1 | BMD | Reference | **0.058 (0.029, 0.087)** | **0.064 (0.036, 0.092)** | **0.069 (0.006, 0.132)*** |
|  | BMC | Reference | **0.852 (0.328, 1.375)** | **0.768 (0.106, 1.431)*** | 1.050 (-0.355, 2.455)* |
|  | Area | Reference | 0.111 (-0.118, 0.339) | 0.002 (-0.507, 0.511)* | 0.206 (-0.536, 0.947)* |
| L2 | BMD | Reference | **0.049 (0.014, 0.084)** | **0.071 (0.034, 0.109)** | **0.092 (0.031, 0.153)*** |
|  | BMC | Reference | 0.597 (-0.036, 1.230) | **0.998 (0.059, 1.937)** | **1.610 (0.360, 2.859)** |
|  | Area | Reference | -0.108 (-0.389, 0.174) | 0.002 (-0.511, 0.514) | 0.255 (-0.339, 0.848) |
| L3 | BMD | Reference | **0.058 (0.022, 0.094)** | **0.077 (0.034, 0.121)** | **0.114 (0.059, 0.170)*** |
|  | BMC | Reference | **0.813 (0.072, 1.553)** | **1.231 (0.013, 2.448)** | **2.140 (0.868, 3.413)*** |
|  | Area | Reference | -0.097 (-0.517, 0.323) | 0.034 (-0.529, 0.597)* | 0.245 (-0.321, 0.811) |
| L4 | BMD | Reference | **0.056 (0.025, 0.087)** | **0.067 (0.019, 0.114)** | **0.095 (0.024, 0.167)*** |
|  | BMC | Reference | **1.088 (0.439, 1.737)** | 0.609 (-0.640, 1.858) | **2.142 (0.375, 3.909)*** |
|  | Area | Reference | 0.140 (-0.280, 0.560) | -0.381 (-0.976, 0.214)* | 0.458 (-0.314, 1.230) |
| **LSM≥11.7** |  |  |  |  |  |
| Total spine | BMD | Reference | 0.097 (-0.013, 0.207) | 0.033 (-0.131, 0.197) | -0.074 (-0.176, 0.028) |
|  | BMC | Reference | 5.907 (-0.909, 12.724) | -3.322 (-15.591, 8.946) | -1.248 (-16.206, 13.709) |
|  | Area | Reference | -1.656 (-5.416, 2.104) | -5.777 (-15.489, 3.935) | 2.572 (-6.943, 12.088) |
| L1 | BMD | Reference | 0.094 (-0.039, 0.227) | -0.008 (-0.193, 0.176) | -0.108 (-0.227, 0.011) |
|  | BMC | Reference | 0.935 (-1.242, 3.112) | -2.140 (-4.851, 0.572) | **-3.028 (-5.251, -0.805)** |
|  | Area | Reference | -0.741 (-1.701, 0.219) | **-2.211 (-3.770, -0.653)** | **-1.502 (-2.732, -0.272)** |
| L2 | BMD | Reference | 0.102 (-0.008, 0.211) | 0.038 (-0.125, 0.202) | -0.026 (-0.117, 0.065) |
|  | BMC | Reference | **2.359 (0.009, 4.709)** | 0.202 (-3.031, 3.435) | -0.670 (-3.226, 1.886) |
|  | Area | Reference | 0.617 (-0.181, 1.414) | -0.287 (-1.698, 1.124) | -0.047 (-1.464, 1.370) |
| L3 | BMD | Reference | **0.126 (0.005, 0.247)** | 0.083 (-0.100, 0.267) | -0.050 (-0.143, 0.042) |
|  | BMC | Reference | 2.135 (-0.579, 4.849) | -0.655 (-4.657, 3.347) | -1.235 (-3.872, 1.401) |
|  | Area | Reference | -0.128 (-0.988, 0.732) | **-1.911 (-3.714, -0.107)** | -0.257 (-1.717, 1.204) |
| L4 | BMD | Reference | 0.143 (-0.046, 0.332) | 0.108 (-0.180, 0.396) | **-0.150 (-0.264, -0.035)** |
|  | BMC | Reference | 2.802 (-1.303, 6.907) | -0.568 (-6.687, 5.551) | -2.355 (-5.239, 0.529) |
|  | Area | Reference | -0.205 (-1.098, 0.689) | **-2.775 (-4.006, -1.544)** | 0.547 (-1.074, 2.169) |

Note: Boldface type indicates statistical significance. An asterisk indicates that the product term of the interaction is significant.

Abbreviations: MASLD: metabolic dysfunction-associated steatotic liver disease; MetALD: metabolic alcohol-related liver disease; ALD: alcoholic liver disease; ‌ LSM: liver stiffness measurement; BMD: bone mineral density; BMC: bone mineral content.
